# Liganded LolCDE structures reveal a common substrate-LolE interaction guiding bacterial lipoprotein transport

**DOI:** 10.1073/pnas.2520579123

**Published:** 2026-01-20

**Authors:** Paul Szewczyk, Nicholas P. Greene, Martyn F. Symmons, Steven W. Hardwick, Vassilis Koronakis

**Affiliations:** ^a^Department of Pathology, University of Cambridge, Cambridge CB2 1QP, United Kingdom; ^b^Department of Biochemistry, University of Cambridge, Cambridge CB2 1GA, United Kingdom

**Keywords:** cryo-electron microscopy, ABC transporter, antibacterial target, Lol lipoprotein trafficking, protein-lipid interactions

## Abstract

Bacterial lipoproteins are key structural components of the outer membrane in Gram-negative bacteria and vital components of machineries required for its biosynthesis and maintenance. The Lol system, essential for viability, directs transport of lipoproteins from the site of biosynthesis on the inner membrane to the outer membrane and has been the target of extensive efforts to develop novel antimicrobial drugs. In the first stage of this transport process, newly synthesized lipoproteins are released from the inner membrane by the ABC transporter LolCDE and passed to the periplasmic chaperone, LolA. Here, we show cryo-EM structures of LolCDE in complex with three different lipoprotein substrates, Lpp, Pal, and LolB, with the latter two bearing a disordered peptide linker between the acyl chains and the globular domain. Our work reveals that when the mature lipoprotein lacks an unstructured linker, the N-terminal portion of the protein is in an unfolded state for transport. The lipoproteins make a sequence-independent but structurally conserved interaction with a cleft on the surface of the periplasmic domain of LolE that promotes efficient transport. We propose a model of lipoprotein export where this interaction acts as pivot point for the peptide portion of the lipoprotein allowing the acyl chains to rotate 180° from their initial position in LolCDE to their binding site in LolA. Our results demonstrate how LolCDE can extrude lipoproteins of diverse sequence and structure and reveal an important detail of a transport process fundamental to bacterial physiology.

## Significance

Lipoproteins in Gram-negative bacteria are vital components of the outer membrane that provides a barrier against antibiotics. An essential transport apparatus, the Lol system, comprises an inner membrane transporter, LolCDE, that extracts lipoproteins from the inner membrane and passes them to the periplasmic chaperone, LolA, for onward trafficking to the outer membrane. Here we show structures of LolCDE in complex with multiple substrates that despite diversity in sequence and structure, share a common site of interaction with a surface cleft of LolE. The interaction is important for efficient lipoprotein export and refines our knowledge of a system that is of fundamental importance to bacterial cell biology and the target of several recently developed antimicrobials.

In Gram-negative bacteria, lipoproteins comprise a class of proteins tethered to the inner or outer membrane by a lipid anchor. They serve a variety of crucial roles in bacterial physiology and pathogen–host interactions including immune avoidance (1), antibiotic detoxification (2, 3), and nutrient acquisition (4). Lipoproteins underpin the structural integrity of the cell envelope (5, 6), and they sense (7), and respond to (8), cell envelope stress. Furthermore, they are essential components of machineries required for maintenance of lipid asymmetry (9, 10) and the insertion of both lipopolysaccharide (11) and outer membrane proteins (12). The synthesis and correct localization of lipoproteins are thus central to the survival of the bacterial cell (13).

Lipoproteins are initially synthesized in the cytoplasm, where an N-terminal signal peptide then directs their translocation across the inner membrane by the Sec or Tat systems (14, 15). Maturation of the lipoprotein is directed by a conserved motif, the lipobox, at the C-terminus of the signal peptide and requires the sequential action of three inner membrane-resident enzymes: Lgt, Lsp, and Lnt. The thiol group of an invariant cysteine in the lipobox is first modified with a diacyl group by Lgt before the signal peptide is removed by Lsp. Finally, a third acyl chain is added to the N-terminal amine of the cysteine by Lnt (16). Some lipoproteins remain inner membrane-resident and possess a transport avoidance motif close to the invariant cysteine (17, 18), but outer membrane lipoproteins require the activity of a dedicated transport apparatus, the Lol system, to achieve correct localization. The Lol system comprises five proteins, LolABCDE. The inner membrane type VII (19, 20) ABC transporter LolCDE exports mature lipoprotein substrates to the periplasmic resident chaperone LolA, which then shuttles them to the outer membrane receptor LolB, itself a lipoprotein, for outer membrane insertion (21–23). LolA recruitment occurs through binding to LolC (24), while LolE has been proposed to recognize segments of the lipoprotein substrate (25). Variant Lol systems in which LolC and LolE are replaced by two copies of a single protein, LolF have been identified (26). In addition, some bacteria may utilize a bifunctional LolA instead of discrete LolA and LolB proteins (27) but in gamma proteobacteria, including **Escherichia coli*,* all five Lol proteins are required for viability under normal conditions (28–30). Dysfunction of the Lol system leads to cell death, principally because of inner membrane retention of Lpp (31). The essential nature of the Lol system, and the lack of any structurally related human homolog, have made it an attractive target for antibiotic development (32–34), including by AI-driven screening of compound libraries (35). Variation between Lol homologues results in differential susceptibility to Lol inhibitors, raising the possibility of therapeutics targeting pathogenic bacteria without affecting resident microbiota (36, 37).

Recent crystallographic and cryoEM studies have established the overall architecture and registration of the LolCDE transporter in multiple conformations, as well as the structural basis for the recruitment of the LolA chaperone (24, 38–41). LolC and LolE possess four transmembrane helices each, with helical extensions of TM1 and TM2 from each monomer forming a four-helix bundle that elevates the periplasmic domains of LolC and LolE above the plane of the membrane, while two copies of LolD comprise the cytoplasmic nucleotide-binding domains (NBDs). The three acyl chains of substrate lipoproteins are bound within two hydrophobic pockets at the interface of the LolC and LolE subunits, but the peptide portions of the substrates were not substantively resolved (38–41). Structures of LolCDE obtained by orthovanadate trapping or using nonhydrolyzable ATP-analogues revealed dimerization of the LolD NBDs, and concurrent approach of the LolC and LolE stalk helices. The resultant collapse of the acyl-chain binding pocket provides a rationale for the expulsion of the lipoprotein triacyl group toward LolA (39–41).

The N-terminal residues of lipoproteins interact with LolE (25, 41) and analysis of lipoprotein sequences revealed an unstructured linker is commonly found between the triacylated cysteine and the globular domain, the presence of which is important for efficient transport of these proteins (42). However, the linker is of variable length with some outer membrane lipoproteins apparently containing little or no linker exemplified by the numerically most abundant *E. coli* protein, Lpp (43). These observations raise questions regarding the role of a disordered linker during lipoprotein transport by LolCDE, how it engages with the transporter, and how lipoproteins lacking a linker are transported. Here, we present structures of *E. coli* LolCDE in complex with three different substrates addressing these questions, and identify a structurally conserved, sequence-independent interaction of the lipoprotein polypeptide with the periplasmic domain of LolE.

## Results

### Structure of Substrate-Engaged LolCDE Complexes.

**Fig. 1.**
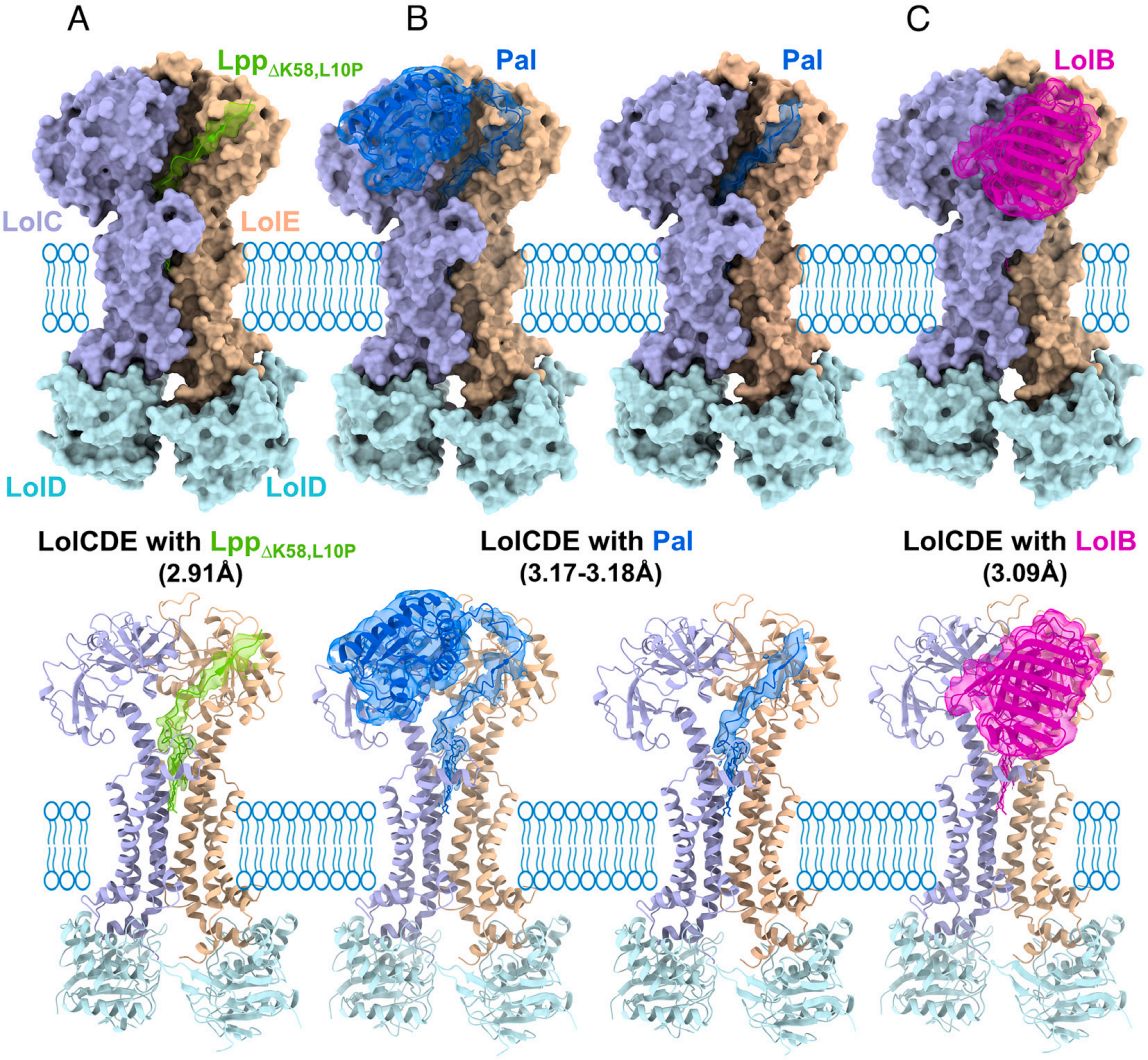
CryoEM models of *E. coli* LolCDE in complex with three different lipoprotein substrates (*A*) LolCDE with Lpp_∆K58,L10P_ at 2.91 Å. (*B*) LolCDE:Pal complex in two states, with the full folded lipoprotein visible in association with LolC at 3.18 Å (*Left*), and the C-terminal globular domain unobserved at 3.17 Å (*Right*). (*C*) LolCDE complexed with LolB fully resolved at 3.09 Å. LolCDE is colored as labeled and shown as space-filled models (*Top*), or ribbons (*Bottom*). The lipoprotein substrates are rendered as ribbon cartoons with a semitransparent map surface overlaid.

Previous structures of LolCDE revealed both the overall architecture of the transporter and the molecular basis for recognition of the lipoprotein triacyl moiety but revealed little detail of the protein moiety of the substrate (38–41). To better understand lipoprotein–transporter interactions, we purified complexes of *E. coli* LolCDE coexpressed with three different lipoprotein substrates diverse in structure and linker length. Lpp is the most abundant *E. coli* lipoprotein with an entirely α-helical structure and no apparent linker (44), while LolB and Pal are predicted to contain unstructured linkers of approximately 15 and 34 residues, respectively (45). Native Lpp contains a C-terminal lysine (Lys58) that crosslinks the outer membrane to peptidoglycan but accumulation of Lpp in the inner membrane results in ectopic crosslinks that lead to cell death (31). To avoid this issue in Lpp overexpressing cells, we utilized both a Lpp variant lacking this C-terminal lysine (Lpp_∆K58_) to permit efficient overexpression and a Lpp_∆K58_ variant containing an additional mutation, L10P (Lpp_∆K58,L10P_), that increases in vitro Lpp transport efficiency by perturbing its trimerization (46). First confirming sample integrity by size exclusion chromatography, and the presence of substrate in purified LolCDE:lipoprotein complexes by immunoblot, (*SI Appendix*, Fig. S1) we then utilized single particle cryoEM microscopy to determine their structures in Lauryl Maltose Neopentyl Glycol (LMNG) at 2.91 to 3.18 Å, with views and major features provided (Fig. 1 and *SI Appendix*, Figs. S2–S4). The cryoEM workflow and data are shown in *SI Appendix*, Figs. S5 and S6 with data collection and refinement statistics shown in *SI Appendix*, Table S1.

All the substrate-engaged LolCDE structures reveal the LolD nucleotide-binding domains (NBDs) are separated and free of nucleotide with the lipoprotein acyl chains bound within pockets at the interface of LolC and LolE. Our 2.91 Å LolCDE:Lpp model represents the highest-resolution structure of this transporter to date, and the overall topology and architecture of our LolCDE structures agree with those recently published in nucleotide-free, substrate-engaged forms derived from nanodisc-reconstituted samples (38, 41) (*SI Appendix*, Table S2, rmsds of 1.52 to 1.54 Å in structural alignments of LolCE). Overall laying between these two models in similarity, differences in our models include the registration and positioning of residues preceding LolE TM1, the N terminus of LolC, the C termini of both LolC and LolE, as well as a repositioning of the Thr245-Ser247 LolE loop. In contrast to previously published data, our maps provide improved coverage of the Lpp peptide that traverses upward in an extended conformation from the triacylated cysteine to the top of the periplasmic domain of LolE. The two Lpp variants exhibited identical conformations other than near the site of the substrate mutation (*SI Appendix*, Fig. S3), and similar resolution (2.91 Å for the Lpp_∆K58,L10P_ model, 3.00 Å for the Lpp_∆K58_ model).

We were able to trace and fit the entire sequences of the Pal and LolB proteins engaged with LolCDE at resolutions of 3.18 Å for LolCDE:Pal, and 3.09 Å for the LolCDE:LolB complex allowing unambiguous identification of the bound substrate. For Pal we obtained an additional complex state at 3.17 Å where only part of the linker was resolved. The Pal globular domain directly contacts the periplasmic domain of LolC, aided by the presence of the C-terminal Strep-tag, although a low-resolution map of LolCDE in complex with untagged Pal reveals the same conformation of the substrate (*SI Appendix*, Fig. S7). In the LolB complex, the folded domain of LolB is located alongside LolE, near the LolC Gly348-Glu361 shoulder loop that connects TM3 and TM4 flanking the presumed substrate entrance portal (38–41). In contrast to Pal, the globular domain of LolB does not make direct contact with the periplasmic domain of the transporter.

### The LolE Periplasmic Domain Specifically Interacts with the Lipoprotein Linker.

**Fig. 2.**
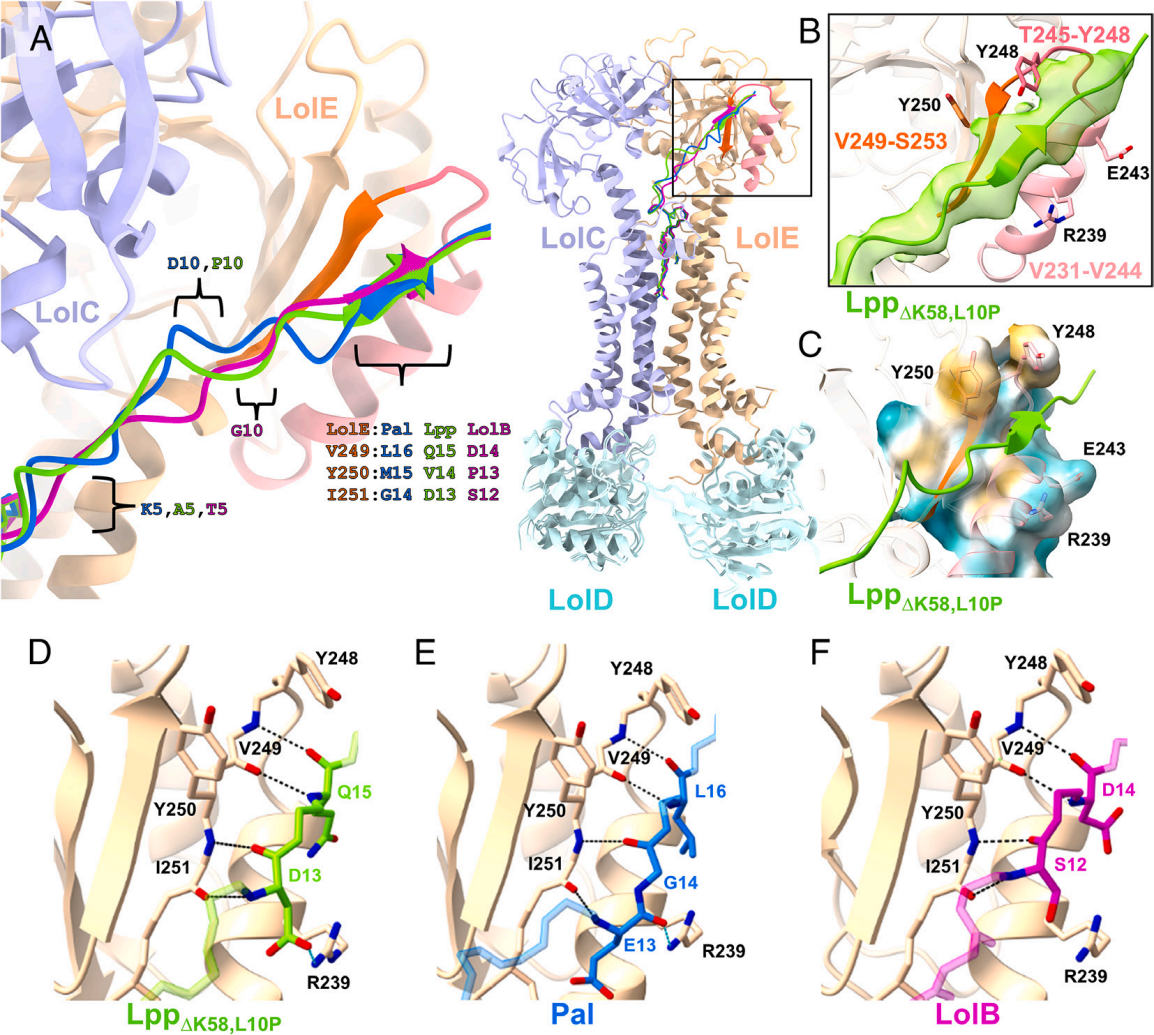
Lipoprotein interaction with a cleft on the periplasmic surface of LolE (*A*) Superposition of lipoprotein linkers and acyl chains from Lpp_∆K58,L10P_ (green), Pal (blue), and LolB (pink). The three residues making up the lipoprotein substrate β-strand (SBS) and registration positions at residues 5 and 10 for each substrate are indicated (*Left*). Ribbon model of LolCDE:Lpp_∆K58,L10P_ with the linkers overlaid (*Right*). (*B*) Close-in view of the boxed region in (*A*). Ribbon cartoon of the LolE cleft showing the residues comprising the short helix (pink), intramolecular joining loop (dark pink), and LolE β-strand (orange). Lpp_∆K58,L10P_ with SβS traversing the cleft is shown in green ribbon, with a 4 Å radius map carve-out centered on the Lpp Cα mainchain overlaid in green. (*C*) Lipophilicity surface map showing the LolE cleft shape, colored from cyan (most hydrophilic), to gold (most lipophilic). Arg239, Glu243, Tyr248, and Tyr250 are shown for orientation with (*B*). (*D*–*F*) Individual ribbon model views of (*C*) Lpp_∆K58,L10P_, (*D*) Pal, and (*E*) LolB traversing the LolE cleft. LolE is shown in cartoon representation with the LolE cleft β-strand and substrate shown in stick representation with potential hydrogen bonds (black), or salt bridges (light blue) indicated by dashed lines. Substrates are colored as in (*A*) and rendered semitransparent as necessary for clarity.

The acyl chains and first three residues of each substrate have near identical conformations (Fig. 2*A*). The subsequent peptide crosses the exterior periplasmic surfaces of LolC and LolE. This stretch of residues exhibits higher sidechain B-factors probably due to the lack of contact with either LolC or LolE but, despite the absence of secondary structure, this region is resolvable within the maps for all three substrates. The trajectories of the peptide chains of the different substrates deviate slightly but all converge at a shallow cleft on the periplasmic face of LolE (Fig. 2*A*) formed between a short helix (Val231-Val244), a joining loop (Thr245-Tyr248), and a β-strand (Val249-Ser253) (Fig. 2*B*). Lipoproteins pass through this cleft bounded by the helix and β-strand, and a clamp consisting of Tyr248 and Tyr250 (*SI Appendix*, Fig. S8 *A*–*D*).

The varying trajectories of the linker sequences across the LolC–LolE interface impact the lipoprotein residue number that contacts the LolE cleft (Fig. 2*A*), but in all cases, three lipoprotein residues largely mediate interaction with the LolE β-strand (Fig. 2 *A* and *D*–*F*) despite the absence of sequence identity in this region of substrate lipoproteins (*SI Appendix*, Fig. S8*E*) (47). The lipoprotein interaction with LolE is sufficient in the case of Lpp_∆K58, L10P_ (Asp13, Val14, and Gln15) to induce formation of a short, substrate β-strand (SβS), a feature absent in fully α-helical, mature, Lpp (44). Residue triplets in Pal (Gly14, Met15, and Leu16), and LolB (Ser12, Pro13, and Asp14) interacting with the LolE β-strand are also resolved as short SβS though the lack of uncomplexed full-length Pal and LolB structures precludes commentary on whether these strands are induced by interaction with LolE. For all three substrates, this interaction is positioned to be stabilized by hydrogen bonds between mainchain elements of LolE Val249 and Ile251 with a donor/acceptor partner from the lipoprotein mainchain as well as buried hydrophobic surface interactions (Fig. 2 *D*–*F*).

A tyrosine pair in LolE, Tyr248 and Tyr250, forms a “clamp” that is positioned to promote hydrophobic interactions with Val14 (Lpp) and Met 15 (Pal) at the central position of the SβS. Finally, in the Lpp-complexed structures, Arg239 within the LolE Val231-Val244 helix, can form an additional hydrogen bond, and salt bridge with the engaged lipoprotein. Interaction of the substrate with the LolE cleft results in an average buried surface area of around 600 Å^2^, with hydrogen bonds and a salt bridge contributing an estimated binding energy of approximately −6 kcal/mol [PISA (48)]. This represents an estimated contribution of approximately 30% of the net binding energy of the complete lipoprotein substrate with LolCDE, with the bulk of the remaining binding energy associated with the acyl chains. Consistent with our structural data, recent crosslinking data demonstrated that three residues, Tyr248, Tyr250, and Ile251 located within this cleft formed site-specific crosslinks with the lipoprotein BamE (*SI Appendix*, Fig. S8*A*) (41).

In the Pal and LolB structures, the entirety of both lipoproteins is resolved allowing the linker region to be fully traced. Following the SβS, the longer Pal linker is more poorly ordered, extending upward, and then bends back toward LolC, terminating at a short helix (Glu35-Asn47) where the Pal globular domain associates with LolC (*SI Appendix*, Fig. S2*C*). In LolB, the far shorter linker spans 15 residues, making a sharp turn at Pro16 into a short helix through Asp24 comprising a portion of LolB’s helical lid (49) before the remainder of the full globular domain is resolved near LolE (*SI Appendix*, Fig. S2*D*). As the sequence register of the LolB domain is known from crystal structures (49), the position of this globular domain corroborates the register of the LolB extended linker. For Lpp, the protein chain extends in an unfolded conformation after the SβS but is unresolved beyond Asn18 precluding commentary on the folded nature of Lpp past this point (*SI Appendix*, Figs. S2*B* and S3 *C* and *D*). However, given mature Lpp has an α-helical structure from the N-terminal triacylated cysteine onward (44), the structure provides further direct evidence that, in lieu of a disordered linker, the mature domain Lpp is transported in an, at least partially, unfolded state.

### Perturbation of Lipoprotein Linker Sequence Identity Does Not Impair Interaction with LolCDE.

**Fig. 3.**
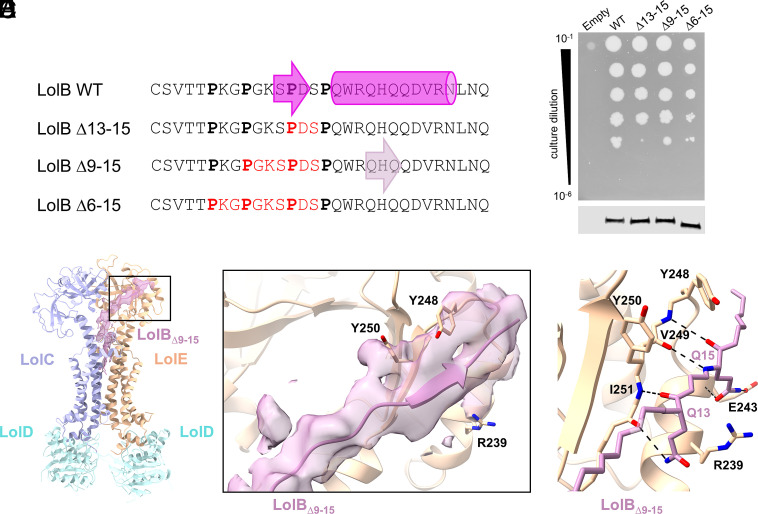
In vivo and structural characterization of LolB variants truncated in the linker region. (*A*) N-terminal sequences of wild-type (WT) and variant LolB with prolines indicated in bold and residues removed colored in red. The position of the SβS (pink arrow) in wild-type LolB and the ∆9-15 variant (mauve arrow), and the LolB N-terminal α-helix (Pro16-Asp24, pink cylinder) are indicated. (*B*) Serial dilutions of a conditional *lolB* knockout *E. coli* strain (BW65) carrying either plasmid-borne wild-type *lolB* or indicated variants on LB agar in the absence of inducer required for expression of chromosomal *lolB* (*Top*). Immunoblot indicating expression of *lolB* wild-type or variants in BW65 cells supported by expression of chromosomal *lolB* (*Bottom*). (*C*) Ribbon model of the LolCDE:LolB_∆9-15_ complex at 3.19 Å with LolB_∆9-15_ ribbon and map colored mauve. (*D*) Zoomed in view of the boxed region in (*C*). LolB_∆9-15_ with SβS traversing the cleft is shown in mauve ribbon, with a 4 Å radius map carve-out centered on the LolB_∆9-15_ Cα mainchain overlaid in mauve. (*E*) Close-up view revealing LolB_∆9-15_ interactions with residues of the LolE cleft, rendered as in Fig. 2 *D*–*F*.

In order to assess positional variance within a given lipoprotein linker, or existence of a cryptic substrate sequence requirement dictating the formation of a SβS with LolE, we generated a series of truncation mutants comprising excisions of proline-flanked residues within the LolB linker (Fig. 3*A*). These excisions encompass the native LolB Ser12-Asp14 SβS residues and necessitate a partial unwinding of the first α-helix of LolB [Pro16-Asp24, (49)] in order to permit the formation of an SβS with LolE Val249-Ile251. LolB localization to the outer membrane is essential for efficient growth. We therefore assayed the LolB variants by expression in a conditional **E. coli* lolB* knockout strain since viability in vivo reflects transport of LolB, or variants, to the outer membrane.

We utilized a plasmid system that expressed Alfa-tagged LolB at a level comparable to that of chromosomally encoded LolB driven by the native LolB promoter (*SI Appendix*, Fig. S9*A*). The mutant LolB proteins were all expressed and could support growth of a conditional *lolB* knockout strain either on unmodified LB agar (Fig. 3*B*), or when challenged with 0.5 mM EDTA and 0.5% SDS in order to assess cell envelope integrity (*SI Appendix*, Fig. S9*B*). Consistent with this, cell fractionation revealed that in all cases, a substantive portion of LolB was localized to the outer membrane although the localization was reduced as severity of the linker deletion increased (*SI Appendix*, Fig. S9*C*). We determined the structure to 3.19 Å of LolCDE in complex with one of the variants (LolB_∆9-15_) that lacks the residues forming the SβS in wild-type LolB, in order to visualize the interaction of this modified substrate with the transporter (Fig. 3 *C* and *D*, data collection/refinement statistics in *SI Appendix*, Figs. S5 and S6 and Table S1). The conformations of LolCDE and the lipoprotein acyl chains are unaffected but the peptide portion of the modified LolB_∆9-15_ substrate adopts a different path toward LolE from the triacylated cysteine residue compared with that in wild-type LolB (Fig. 3*D*). However, crucially, the variant lipoprotein still interacts with the LolE cleft mediated by an SβS, in this case formed by residues Gln13, His14, and Gln15 (residues 20 to 22 of the wild-type sequence), a change in registration from the Ser12, Pro13, and Asp14 triplet SβS in wild-type LolB (Fig. 2 *A* and *F*). As in the wild-type LolB complex, LolB_∆9-15_ forms interactions with LolE Val249 and Ile251, in this case mediated by Gln13 and Gln15 (Fig. 3*E*). However, in the truncated variant, the sidechain of Gln15 is positioned to form additional bonds with the mainchain and sidechains of LolE Glu243 underscoring the adaptability of lipoprotein binding within the LolE cleft. We resolved the LolB_∆9-15_ sequence as far as residue Asp17 (corresponding to Asp24 in the wild-type sequence) confirming that the N-terminal helix of mature LolB (wild-type residues 19 to 26) must be unwound. The additional freedom allowed by this abolition of secondary structure is likely the reason that, in this case, the rest of the LolB globular domain was not resolved. EM reconstructions generated no states in which the truncated protein failed to interact with the LolE cleft. Overall, our data underline the absence of specific sequence requirement and demonstrate an inherent flexibility in formation of the SβS with the LolE cleft. Characterization of the LolB variants reveal that, as for Lpp, in the absence of an unstructured linker, α-helical elements of the mature lipoprotein can engage with the LolCDE transporter in an unfolded state to permit transport.

### Probing the Importance of the Substrate Interacting Region of LolE.

**Fig. 4.**
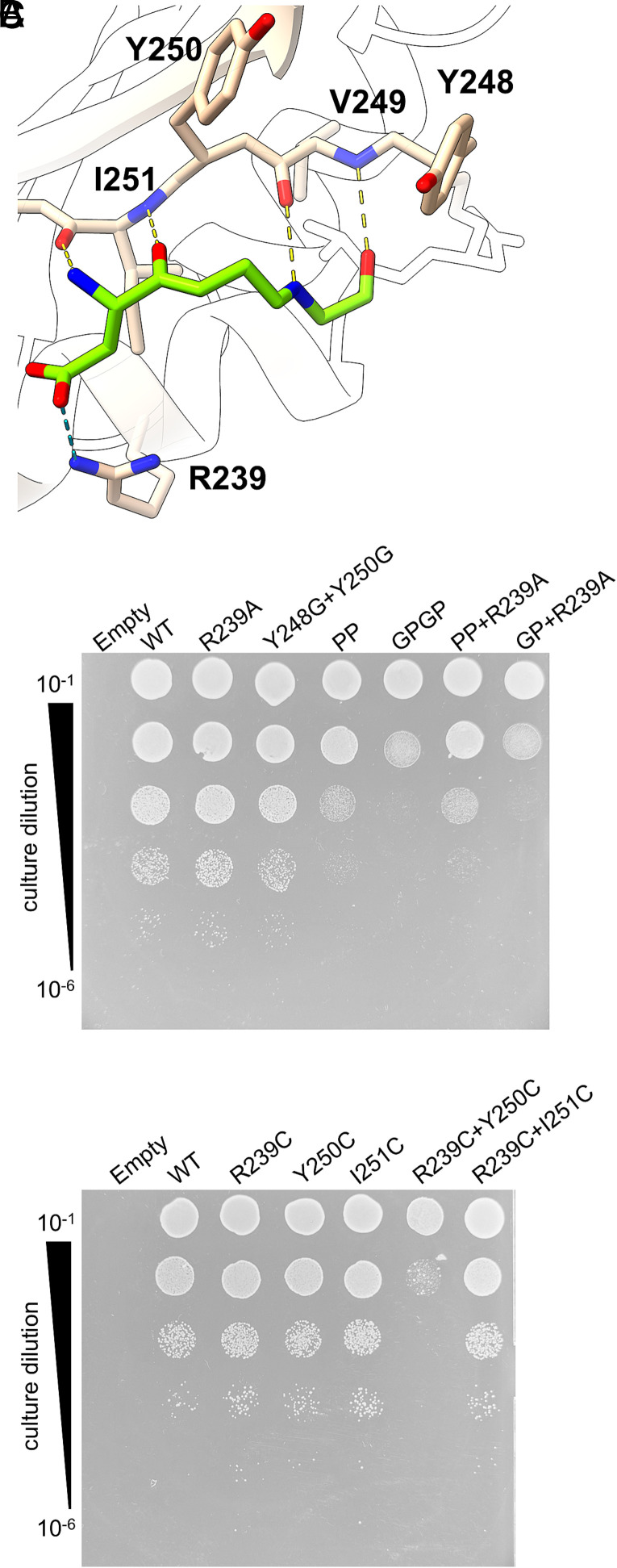
In vivo analysis of mutations in the LolE cleft (*A*) Cartoon view of the LolE cleft from the LolCDE:Lpp_∆K58,L10P_ complex with LolE residues targeted for mutagenesis shown as tan sticks. (*B*) Serial dilutions of a conditional *lolCDE* knockout *E. coli* strain (HD200313) carrying either plasmid-borne wild-type *lolCDE* or indicated variants in the absence of inducer required for expression of chromosomal *lolCDE.* Plates were supplemented with 0.5 mM EDTA and 0.5% SDS. (*C*) Serial dilutions of a conditional *lolCDE* knockout *E. coli* strain (HD200313) carrying either plasmid-borne wild-type *lolCDE* or indicated cysteine variants in the absence of inducer required for expression of chromosomal *lolCDE.* Plates were supplemented with 0.5 mM EDTA and 0.5% SDS.

We next investigated the effect of mutating individual cleft residues that were involved in the interaction with lipoprotein substrate (Fig. 4*A*). Using a conditional *lolCDE* knockout strain (40) we monitored the ability of these variants to support growth on either unmodified medium or that containing EDTA and SDS. Growth in the presence of arabinose to induce expression of the chromosomal wild-type *lolCDE* demonstrated the viability of the cells and, in the absence of arabinose, growth could be complemented by plasmid-borne *lolCD_His_E* (*SI Appendix*, Fig. S10*A*). Simultaneous glycine substitution of LolE Tyr248 and Tyr250, which form a clamp around the central methionine or valine residues of the SβS in Pal and Lpp, respectively, did not impact growth on plain medium (*SI Appendix*, Fig. S10*A*) or that containing EDTA and SDS (Fig. 4*B*). Similarly, alanine substitution of LolE Arg239 that interacts with Lpp had no appreciable effect and the growth of neither mutant was significantly different in liquid culture to cells expressing wild-type LolCDE (*SI Appendix*, Fig. S10*B*). As the β-strand interaction is underpinned by mainchain hydrogen bonds (Figs. 2 *D*–*F* and 4*A*), we introduced prolines into LolE to eliminate hydrogen bond donors at positions V249 and I251 (LolE_PP_). This yielded a modest growth impairment in the presence of SDS/EDTA stress that was exacerbated by concomitant mutation of Tyr248 and Tyr250 to glycine (LolE_GPGP_; Fig. 4*B*). Consistent with this, both LolE_PP_ and LolE_GPGP_ variants exhibited reduced growth rates in liquid culture (*SI Appendix*, Fig S10*B*). The introduction of the R239A mutation into LolE_PP_ or LolE_GPGP_ had no additional effect on growth. As the LolCDE_PP_ mutant might still allow the formation of hydrogen bonds between the lipoprotein substrate and backbone carbonyl elements of the LolE cleft within this region, we attempted to eliminate the substrate mainchain hydrogen bond donors by concomitant introduction of prolines into Lpp at positions Q13 and D15. These proline variants were able to support the growth of an *lpp* deletion strain on medium containing SDS/EDTA to the same extent as plasmid-borne wild-type *lpp* (*SI Appendix*, Fig. S10*C*). suggesting that Lpp found alternate points of contact with LolCDE consistent with the plasticity of the substrate–cleft interaction as previously observed for the LolB linker truncation (Fig. 3*E*).

We next introduced cysteine point mutants into the LolE cleft with the aim that adducts, or opportunistic crosslinks in vivo, would sterically block interaction of the substrate SβS with the cleft. We therefore created single cysteine variants at positions Arg239 on the cleft helix and at two positions, Tyr250 and Ile251 on the cleft β-strand. Substitution for individual cysteine residues at LolE positions 239, 250, and 251 or simultaneous substitution of Arg239 and Ile251 did not affect growth but simultaneous mutation of Arg239 and Tyr250 (hereafter LolCDE_cc_) to cysteine resulted in a modest decrease in liquid culture growth rate (*SI Appendix*, Fig. S11), and a significant decrease in viability when cells were grown under EDTA/SDS stress (Fig. 4*C*) with controls provided in *SI Appendix*, Fig. S11.

**Fig. 5.**
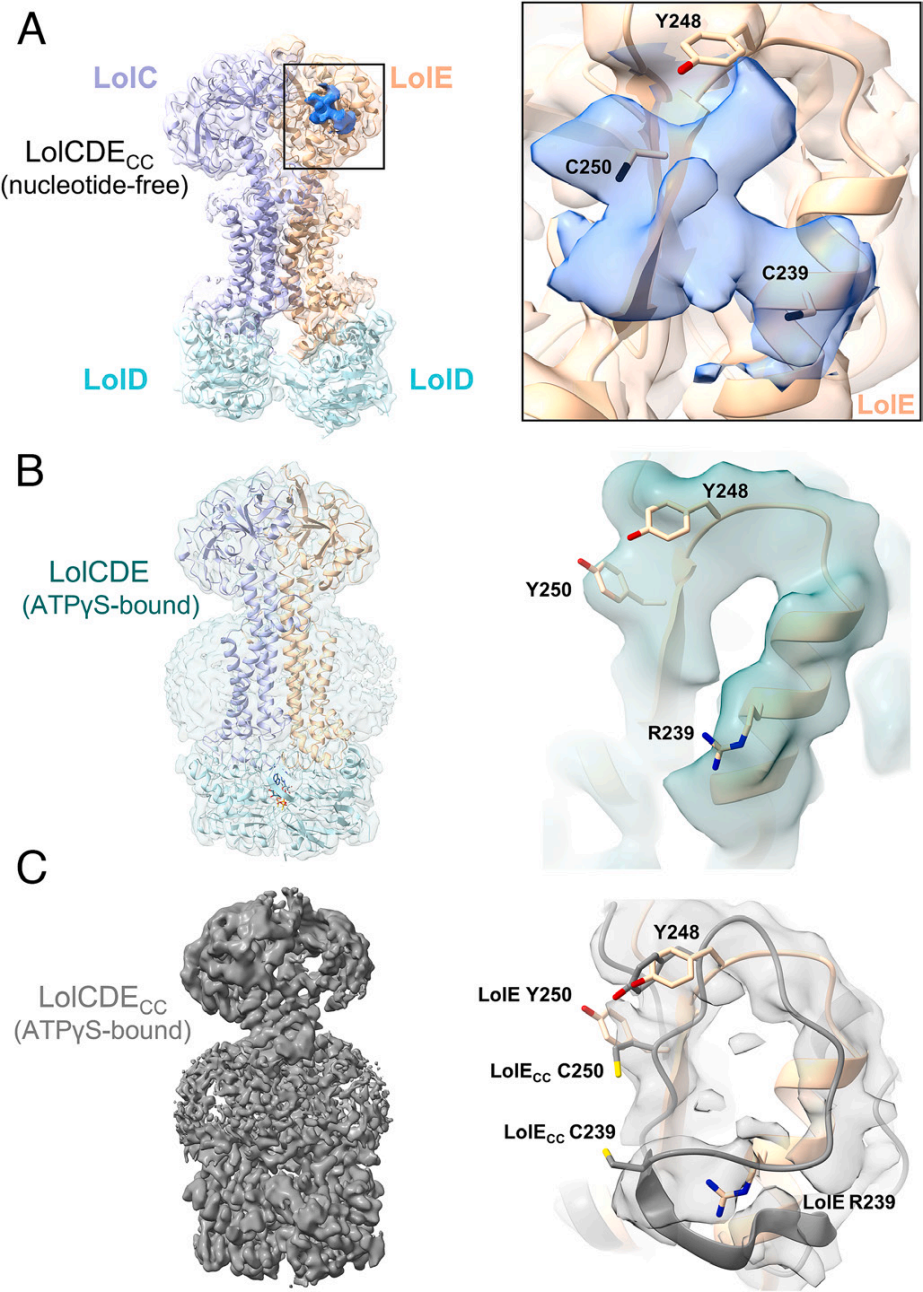
Interrogating LolCDE–substrate interactions using cysteine variants of residues in the LolE cleft. (*A*) Structure of the LolCDE_CC_:Pal complex at 3.48 Å (*Left*), with a close-in view of the boxed region showing the cross-shaped density in blue (*Right*). Residues Cys239, Tyr248, and Cys250 are shown in stick representation. (*B*) Model of ATPγS-bound wild-type LolCDE at 2.9 Å shown in ribbon format with map surface in transparent pale green (*Left*). Close-up view of the LolE cleft, the positions of residues Arg239, Tyr248, and Tyr250 indicated (*Right*). (*C*) Map of ATPγS-bound LolCDE_CC_ at 3.26 Å (*Left*). Close-up view showing the map in the cleft region (pale gray surface) and a speculative trace of the LolCDE_CC_ cleft (gray ribbon), with LolE, from the wild-type ATPγS-bound LolCDE structure, superpositioned (tan). Locations of the residues at positions 239, 248, and 250 in both wild-type LolCDE and LolCDE_CC_ are shown in stick format (*Right*).

In our substrate bound models LolE Arg239 and Tyr250 lie ~11 Å apart precluding any disulfide linkage in this state. To understand the basis for growth inhibition, we therefore generated a 3.48 Å map of LolCDE_cc_ coexpressed with Pal by cryoEM (Fig. 5*A*). Maps of this structure reveal the lipoprotein acyl chains clearly visible within the binding pocket but also show the presence of cross-shaped density situated toward the end of the cleft. Two opposing arms of this cross overlay the position of the introduced cysteine residues, LolE Cys239 and Cys250, though additional density suggests they have been modified in some way (Fig. 5 *A*, *Right*). Mass spectrometry of the purified protein failed to identify the nature of any adduct and structural analysis of the additional noncysteine covering “cross” density is further complicated by the possibility of linker contributions to the map along the remaining two orthogonal cross-arms. However, we resolved neither any linker past the cross nor the globular domain of Pal. In order then to discriminate between potential modifications of the introduced cysteines, and lipoprotein linker we added the nonhydrolyzable ATP analogue, ATPγS, to LolCDE:Pal and LolCDE_CC_:Pal to generate transporter samples in a ligand-free state.

Maps of LolCDE_ATPγS_, and LolCDE_CC:ATPγS_, were generated at 2.9 Å, and 3.26 Å (Fig. 5 *B* and *C*, data collection/refinement statistics in *SI Appendix*, Figs. S5 and S6 and Table S1). As with the open state structures, the overall core architecture of our fitted closed state structures is again similar to those recently published, however there are numerous changes in the placement of loops throughout the models [rmsd 3.23 to 3.6 Å, (38, 41) *SI Appendix*, Table S2]. These changes may reflect our higher-resolution data, local changes between differentially reconstituted samples in detergent versus nanodiscs, or a combination of both. Aligning any of our nucleotide-free LolCDE:lipoprotein models with that of LolCDE_ATPγS_ by LolE TM2, or the LolE cleft shows that lipoprotein linkers could maintain an interaction with the LolE cleft following expulsion of the acyl chains from the central cavity (*SI Appendix*, Fig. S12), although we did not observe any lipoprotein density in our LolCDE_ATPγS_, or LolCDE_CC:ATPγS_ maps.

Compared with the closed wild-type protein (LolCDE_ATPγS_), LolCDE_CC:ATPγS_ map coverage within the cleft is less complete but a speculative build suggests a distortion of the LolE 231 to 244 cleft helix that brings the partially unraveled helix closer to the LolE β-strand thereby narrowing the cleft (Fig. 5 *C*, *Right*). Cysteines at positions 239 and 250 would still be separated by ~6 Å, too far apart to form a disulfide bond but in the oxidizing environment of the periplasm, a range of complex modifications are possible including oxidation to sulfinic or sulfonic acids (50), or formation of adducts between lysines and one or more cysteines (51, 52). Although the nature of the modification remains unresolved, our analysis of the cysteine variants again indicates that modification of the LolE cleft interferes with efficient lipoprotein transport.

### Removal of the LolE Cleft Severely Impacts LolCDE Function in Wild-Type Cells.

**Fig. 6.**
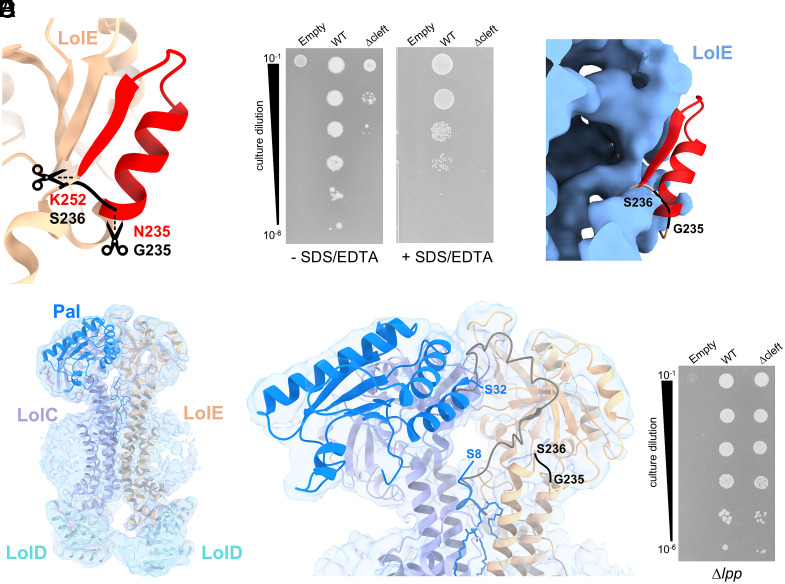
Analysis of a LolE variant lacking the substrate interaction cleft. (*A*) Schematic of LolE indicating the replacement of residues 235 to 252 (red) with GS in the LolE_∆cleft_ variant. (*B*) Serial dilutions of the conditional *lolCDE* knockout strain, (HD200313), carrying either plasmid-borne wild-type *lolCDE* or *lolCDE_∆_*_cleft_were grown in the presence, or absence, of 0.5 mM EDTA/0.5% SDS. (*C*) Zoomed in view of the LolE periplasmic domain of the LolCDE_∆cleft_ map with the position of the excised cleft secondary structure elements (residues 235 to 252) in wild-type LolCDE indicated in red. The GS linker used to replace the cleft structure is indicated in black. (*D*) Structure of the LolCDE_∆cleft_:Pal 3.63 Å complex with the model in cartoon representation and the map indicated in transparent blue surface. (*E*) Zoomed in view of the LolCDE_∆cleft_:Pal complex shown in (*D*). The GS linker that replaces the LolE cleft is indicated in black. The limits of the resolved Pal linker are indicated while the path of the Pal linker in the wild-type LolCDE: Pal complex is shown in dark gray. (*F*) Serial dilutions of HD200313 ∆*lpp* carrying either plasmid-borne wild-type *lolCDE* or *lolCDE_∆_*_cleft_as indicated.

The flexibility of the interaction between the lipoprotein protein chain and the LolE cleft complicated analysis of point mutations in this region. Therefore, to understand the importance of this interaction for LolCDE-mediated transport, we replaced LolE residues 235 to 252 with a GlySer linker thereby removing the β-strand, interconnecting loop, and short helix forming the LolE cleft (Fig. 6*A*). The variant lacking these structural elements, LolE_∆cleft_, exhibited significantly impaired growth in the absence of stress and was not viable in the presence of SDS/EDTA, despite being expressed and integrated into the membrane (Fig. 6*B* and *SI Appendix*, Fig. S13 *A* and *B*) To visualize the effect of the cleft deletion on the conformation of LolE, we solved the structure of LolCDE_∆cleft_ in complex with Pal (Fig. 6 *C*–*E*), with data collection and refinement statistics in *SI Appendix*, Figs. S5 and S6 and Table S1.

As with the wild-type LolCDE:Pal complex, purified protein was monodisperse (*SI Appendix*, Fig. S13*C*) and resolved by cryoEM as two states (at 3.63 Å and 3.29 Å, respectively) in which the globular domain of Pal was either evident (Fig. 6*D*) or not resolved (*SI Appendix*, Fig. S13 *D* and *E*). The variant LolE is properly folded (rmsd of 0.873 Å across 176 Cα atoms from residues 58 to 234 between LolE_∆cleft_, and LolE). In both states, the acyl chains and the first 7 to 8 residues of Pal are resolved, demonstrating that the variant LolE is still competent to accommodate the triacyl moiety. In addition, the globular domain of Pal is resolved in the same location as in the wild-type LolCDE:Pal complex. However, in both states of the LolCDE_∆cleft_ complex, the linker region (residues 9 to 32) is poorly ordered and lacks close association with LolE thus underlining the requirement of the LolE cleft for interaction with the lipoprotein chain (Fig. 6*E* and *SI Appendix*, Fig. S13 *D* and *E*). We reasoned that low viability of cells expressing the LolE_∆cleft_ variant was likely due to inefficient Lpp transport (31). We therefore deleted *lpp* from the conditional LolCDE knockout strain and found that in this background, the variant was able to complement growth in the absence of stress (Fig. 6*F* and *SI Appendix*, Fig. S13*F*). Taken altogether the in vivo growth data and the complex structure demonstrate that the interaction of the peptide portion of the lipoprotein with LolE is important for efficient lipoprotein transport.

## Discussion

Here, we report structures illustrating the engagement of three distinct lipoproteins, Lpp, Pal, and LolB, with the LolCDE transporter. Previous work (38–41) revealed the overall topology and fold of LolCDE, but incomplete coverage outside the acyl chains of any engaged substrate. Our structures reveal the full binding modalities of Pal, and LolB, while expanding on that of Lpp. The acyl chains of all substrates overlap in common binding pockets; however, each substrate differs in its trajectory across the intratransporter LolC–LolE interface before converging at a cleft on the periplasmic face of LolE. This cleft, encompassing the periplasmic exterior surface of LolE residues Ser231-Val253, comprises a short α-helix, loop, and β-strand which immediately precedes LolE TM2. As substrate traverses this cleft, a short, lipoprotein substrate β-strand (SβS) can be observed stabilized by LolE-substrate intermolecular hydrogen bonds from LolE Val249 and Ile251, with potential contributions from the Tyr248-Tyr250 clamp, and/or side and main chain interactions with residues of the Val231-Val244 helix. Our data demonstrate that three distinct substrates form this interaction while unnatural amino acid crosslinking data suggest that a fourth lipoprotein, BamE, likely has equivalent interactions with LolE (41). We therefore believe it is likely that the interactions we observe are a common feature of lipoprotein engagement with LolCDE.

Our structures revealed the first full-length structures of triacylated Pal and LolB which allowed unambiguous identification of these proteins. In the Pal complex, we observed direct contact of the globular domain of the mature lipoprotein with the LolCDE complex. This interaction is mediated by the C-terminal Strep-tag but also occurs when the tag is absent (*SI Appendix*, Fig. S7). While it may not be physiological, sequestering the globular domain of lipoproteins through low affinity interactions with the transporter could conceivably prevent ectopic interactions with partner proteins prior to outer membrane localization.

The interaction of substrate lipoproteins with the LolE cleft is not strictly dependent on sequence as exemplified by our LolB structures where removal of a section of the linker surprisingly results in an adjacent region of LolB adapting to form an interaction with the LolE cleft (Fig. 3). There are few structures of triacylated full-length lipoproteins but interestingly, a recent structure of the outer membrane lipoprotein SlyB reveals a short β-strand at residues 10 to 12 (8) which would be in prime position to interact with the LolE cleft during export. An interaction of a short induced β-strand from unstructured peptide during protein transport has also been observed in SecA-mediated protein export across the bacterial inner membrane (53, 54) suggesting that this may be a more common feature of protein translocation than previously anticipated.

Our Lpp structures strongly suggest that when a lipoprotein lacks a linker between the acyl chains and any globular domain, it can be recognized and transported by LolCDE in at least a partially unfolded state. Periplasmic chaperones such as Skp and SurA are not apparently essential for the export of lipoproteins (55), and it may be that Lpp, which has relatively slow folding kinetics (56, 57) remains in a transport competent unfolded state during the maturation of the lipoyl moiety on the inner membrane. Alternatively, but less likely, LolCDE could play a role in actively unfolding the N terminus of the lipoprotein. In either case, lack of helical structure would prevent trimerization of Lpp on the inner membrane which would likely complicate subsequent export. These observations are consistent with the fact that introduction of a proline residue at position 10, which would presumably retard adoption of helical structure, maintains a transport competent state of this variant for longer than wild-type Lpp (46).

A previous study (42) highlighted the importance of unstructured linkers for LolCDE-mediated transport, but their observations would seem at odds with the absence of a linker in the highly abundant lipoprotein, Lpp, whose proper and timely transport is essential to bacterial viability (31). This situation is remedied by the observation of Lpp engagement with LolCDE in an unfolded state. The presence of an unstructured linker may partly reflect the function of the lipoprotein; for example, NlpE must reach across the periplasm to interact with CpxA on the inner membrane (58) or to allow RcsF and other lipoproteins to thread through outer membrane proteins to reach the cell surface (7, 59). However, it may also reflect variation in the folding rate or the difficulty in maintaining different lipoproteins in a transport-competent, unfolded state. We demonstrate that an unwound N-terminal helix allows LolB to retain a transport competent state even after the removal of more than half of its linker (Fig. 3), albeit with reduced outer membrane localization (*SI Appendix*, Fig S9*D*). Similarly, truncation of the linkers in Pal, RcsF, and NlpD (42) still appears to allow outer membrane localization of a proportion of the variant lipoprotein. We speculate that transport in these cases occurs at a reduced rate. Structures of full-length lipoproteins are relatively scarce, but the conjugation surface exclusion protein TraT (60), like Lpp, lacks a linker, and possesses secondary structural elements at the extreme N terminus that would have to be maintained in an unfolded state to permit transport.

**Fig. 7.**
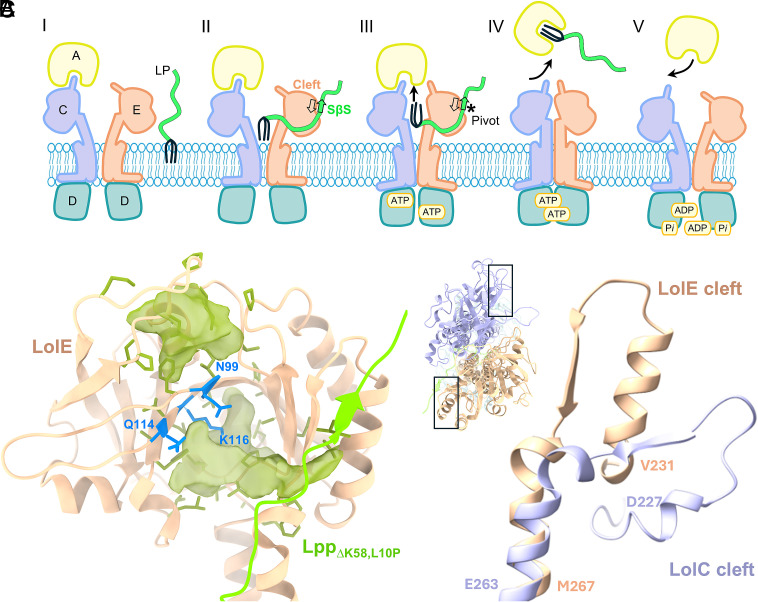
The role of the LolE cleft in lipoprotein extrusion by LolCDE. (*A*) Model of lipoprotein extraction by LolCDE. (i) LolA binds LolCDE in the open, nucleotide-free state while the lipoprotein is still unfolded (ii) Triacyl groups of the substrate bind the pocket at the LolC/LolE interface and the unfolded N-terminal residues of the lipoprotein form an SβS upon engagement with the LolE cleft. (iii) Binding of ATP induces the closure of the transporter transmembrane helices first, which elevates the lipoprotein in a cavity formed by the LolC and LolE periplasmic domains forcing the acyl chains to rotate in this cavity by 180° clockwise around an axis running from LolC to LolE. The cleft interaction can act as a pivot point to restrain the protein moiety of the substrate during this rotation. (iv) Lipoprotein acyl chains are transferred to the LolA cavity, dislodging the LolC Hook. (v) Hydrolysis of ATP then resets the system allowing another cycle to begin. (*B*) View of the LolE periplasmic domain from the LolCDE:Lpp_∆K58,L10P_ complex. Internal cavities (green surface) with hydrophobic residues within 3.5 Å are shown as green sticks. Residues forming the polar ledge between the two cavities are shown as blue sticks. Lpp is shown as bright green cartoon. Cavities determined with pyKVFinder (61). (*C*) Alignment of the LolE cleft with the topologically equivalent cleft region of LolC. LolE residues V231-M267 and LolC D227-E263 are shown. Top–down view of the transporter with regions of the compared clefts boxed (*Inset, Top Left*).

Our data allow us to refine the model of lipoprotein export by LolCDE (Fig. 7*A*). The lipoprotein engages the LolCDE transporter through interactions with its acyl chains and the SβS. The triacyl moiety binds at the transmembrane interface of LolC and LolE with the acyl chains pointing toward the cytoplasm and ultimately must reach the chaperone LolA at the top of the LolC periplasmic domain. The orientation of LolA on LolCDE (24) and of the acyl chains within LolA (47) means that the acyl chains must rotate 180° within the periplasmic domains of LolCE during the course of this translocation. In the open state, LolE contains two largely hydrophobic cavities with an intervening “ledge” of polar residues (Asn99, Q114, and K116), with the lower pocket extending to the SβS (Fig. 7*B*). These pockets could serve as the docking site of the acyl chains as they are expelled from their initial binding sites following nucleotide binding, and the closure of LolE and LolC TMs 1 and 2. We speculate that the LolE:SβS interface serves as a pivot during transport, holding the main body of the lipoprotein substrate away from the interior of LolCDE, and restraining the movement of the acyl chains by constraining the number of residues between the periplasmic LolE cavity and the LolE:SβS pivot, thereby promoting efficient transport. In the absence of the cleft, transport is still possible but at an insufficient rate to prevent toxic build-up of Lpp on the inner membrane (Fig. 6). As we do not observe a LolE:SβS interaction in the ATPγS-bound LolCDE map, disruption of the LolE:SβS interaction must occur following acyl chain loading into LolA, and displacement of loaded LolA from LolCDE.

Further work is required to understand how the LolE:SβS interaction would be abrogated during transport and whether the interface could be targeted with β-strand mimetics as has been proposed for the Bam system (62). In *E. coli*, LolC and LolE share broad structural features but have specialized functions relating to LolA and lipoprotein recognition, respectively (24, 25). The analogous cleft in LolC on the opposite face of the transporter is less structured, with a far shorter β-strand and lacking a substantive α-helix, imposing an additional level of asymmetry on the opposing faces of the transporter (Fig. 7*C*). It will therefore be interesting to see how and if this interaction is preserved in bacterial species which utilize a homodimeric LolDF type transporter in which lipoprotein loading during transport might occur essentially across either symmetric face.

## Methods

Complete methods are available in *SI Appendix, Supplementary Methods*. Briefly, LolCDE:lipoprotein samples for cryoEM were generated by overexpression in *E. coli* C43 cells (63). Protein complexes were purified from isolated membranes solubilized with Lauryl Maltose Neopentyl Glycol (LMNG, Anatrace) utilizing Promega IMAC (Biorad), Strep-Tactin 4Flow (IBA), or ALFA Selector CE (NanoTag) resin as needed. Samples were concentrated to ~500 µL in a 100 kDa cutoff Amicon-Ultra 15 spin filter (Millipore) and injected onto a Superdex 200 10/300GL gel filtration column at 4 °C, with samples for cryo-EM taken as the mid-peak fraction of 200 µL separation aliquots. Where required, 5 mM ATPγS was added to samples to generate the closed form of the transporter. Samples were deposited onto glow-discharged Quantifoil Cu 300 1.2/1.3 grids and blotted with a blot force of −5 for 3 s using a Vitrobot Mark IV (ThermoFisher) at 4 °C and 95% humidity immediately before plunge-freezing in liquid ethane. Datasets were collected on a 300 kV Titan Krios (ThermoFisher) equipped with a Gatan K3 or Falcon 4i detector. Particles were picked and preprocessed (motion correction, CTF estimation, and particle extraction) with WARP1.0.9 (64), and particle stacks migrated to cryoSPARC V4.0 (65). All maps were anisotropically sharpened as implemented within PHENIX (66) and deposited to the EMDB. LolCDE models were built in COOT (67, 68) and refined in PHENIX (66) initially guided by PDB 7ARH, 7ARI, and 7ARJ. The folded domain of LolB was built using PDB 1IWM (49) and that of Pal using PDB 2W8B (69) and PDB 4PWT. Mutations in LolCDE or LolB were introduced by Quikchange mutagenesis (Agilent) and confirmed by DNA sequencing (Source Bioscience). A conditional *lolCDE* knockout strain (HD200313) with the native *lolCDE* under the control of an arabinose inducible promoter (40) was used to assess activity of plasmid-borne *lolCDE* variants either in liquid culture in 48-well plates using a CLARIOstar Plus Plate reader (BMG Labtech) or on LB-agar plates. Where required, agar plates were supplemented with 0.5 mM EDTA and 0.5% SDS to reveal a compromised cell envelope. The *lpp* gene was removed from strain HD200313 using the λ Red recombinase system (70) and plasmid pSIM5 for recombinase expression. A *lolB* conditional knockout strain, BW65, was created by inserting the *lolB* allele under the control of an arabinose promoter at the lambda attachment site and removing the native gene by lambda red recombination (70). This strain was then used to monitor the ability of plasmid-borne variants of LolB to support growth in the absence of arabinose and, following sucrose gradient fractionation, used to assess the proportion of LolB transported to the outer membrane. Molecular graphics and analyses were performed with UCSF ChimeraX (71), developed by the Resource for Biocomputing, Visualization, and Informatics at the University of California.

## Supplementary Material

Appendix 01 (PDF)

## Data Availability

Structures were deposited in the Protein databank (PDB) and Electron Microscopy databank (EMDB) with accession codes PDB 9RLC (72) and EMD-54033 (73) (LolCDE:Lpp_∆K58,L10P_), PDB 9RLD (74) and EMD-54034 (75) LolCDE:Lpp_∆K58_), PDB 9RLE (76) and EMD-54035 (77) (LolCDE:Pal), PDB 9RLF (78) and EMD-54036 (79) (LolCDE:Pal linker only), PDB 9RLG (80) and EMD-54037 (81) (LolCDE:LolB), PDB 9RLH (82) and EMD-54038 (83) (LolCDE:LolB_∆9-15_), PDB 9RLJ (84) and EMD-54040 (85) (LolCDE:ATPγS), PDB 9RLI (86) and EMD-54039 (87) (LolCDE_∆cleft_:Pal), PDB 9RLK (88) and EMD-54041 (89) (LolCDE_∆cleft_:Pal linker only). Maps for Pal-complexed LolCDE_CC_ in the closed and open form were deposited in the EMDB with accession codes EMD-54478 (90) and EMD-54479 (91).
