# Supplementary material for: Liganded LolCDE structures reveal a common substrate-LolE interaction guiding bacterial lipoprotein transport
Source: Proc Natl Acad Sci U S A. 2026 Jan 20;123(4):e2520579123. doi: 10.1073/pnas.2520579123 (PMC12846838; doi:10.1073/pnas.2520579123)
Supplement: Supplementary file 1 — Appendix 01 (PDF) [file pnas.2520579123.sapp.pdf]

Supporting information for:

Liganded LolCDE structures reveal a conserved substrate-LolE interaction guiding bacterial lipoprotein transport.

Paul Szewczyk[*1], Nicholas P Greene[*1], Martyn F Symmons[1], Steven W Hardwick[2], Vassilis Koronakis[†1]

[1]Department of Pathology, University of Cambridge, Cambridge CB2 1QP, United Kingdom

[2]Department of Biochemistry, University of Cambridge, Cambridge CB2 1GA, United Kingdom

[†]Corresponding author: vk103@cam.ac.uk

*Contributed equally to this work

This PDF file includes:

Figures S1 to S13
Tables S1 to S4
Supplemental Methods
SI References

Supplementary Figures

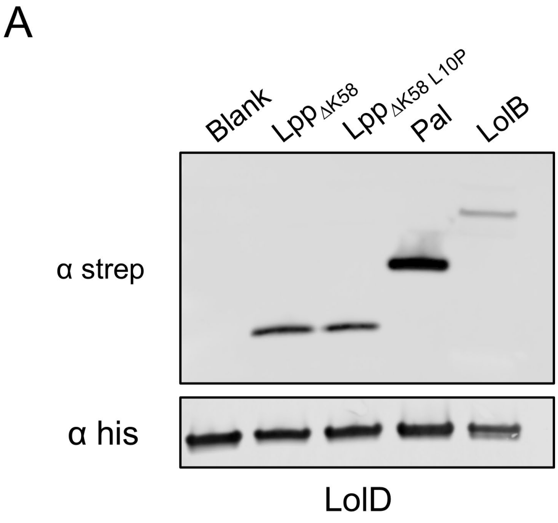

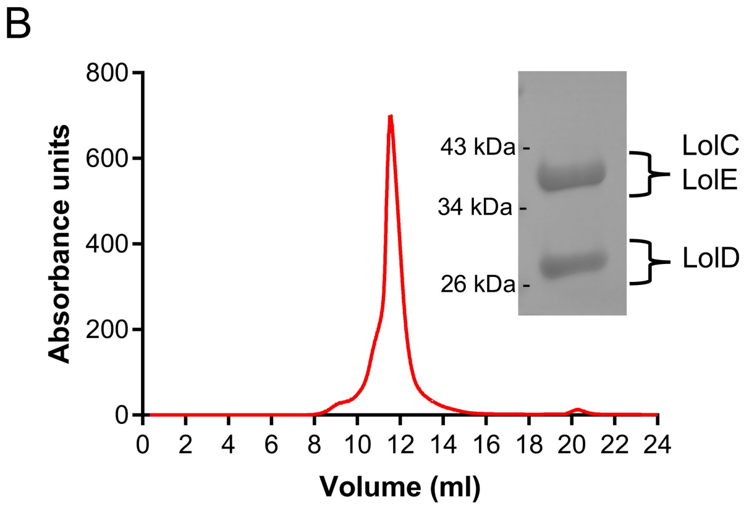

Fig. S1. Expression of LolCDE-substrate complexes. (A) Purified complex was immunoblotted with anti-Strep (*upper panel*) and anti-His (*lower panel*) to indicate the presence of Strep-tagged substrate and His-tagged LolD, respectively. (B) Size exclusion chromatography trace of purified $Lpp_{\Delta K58,L10P}$ (*left*) with SDS-PAGE of same shown boxed (*right*).

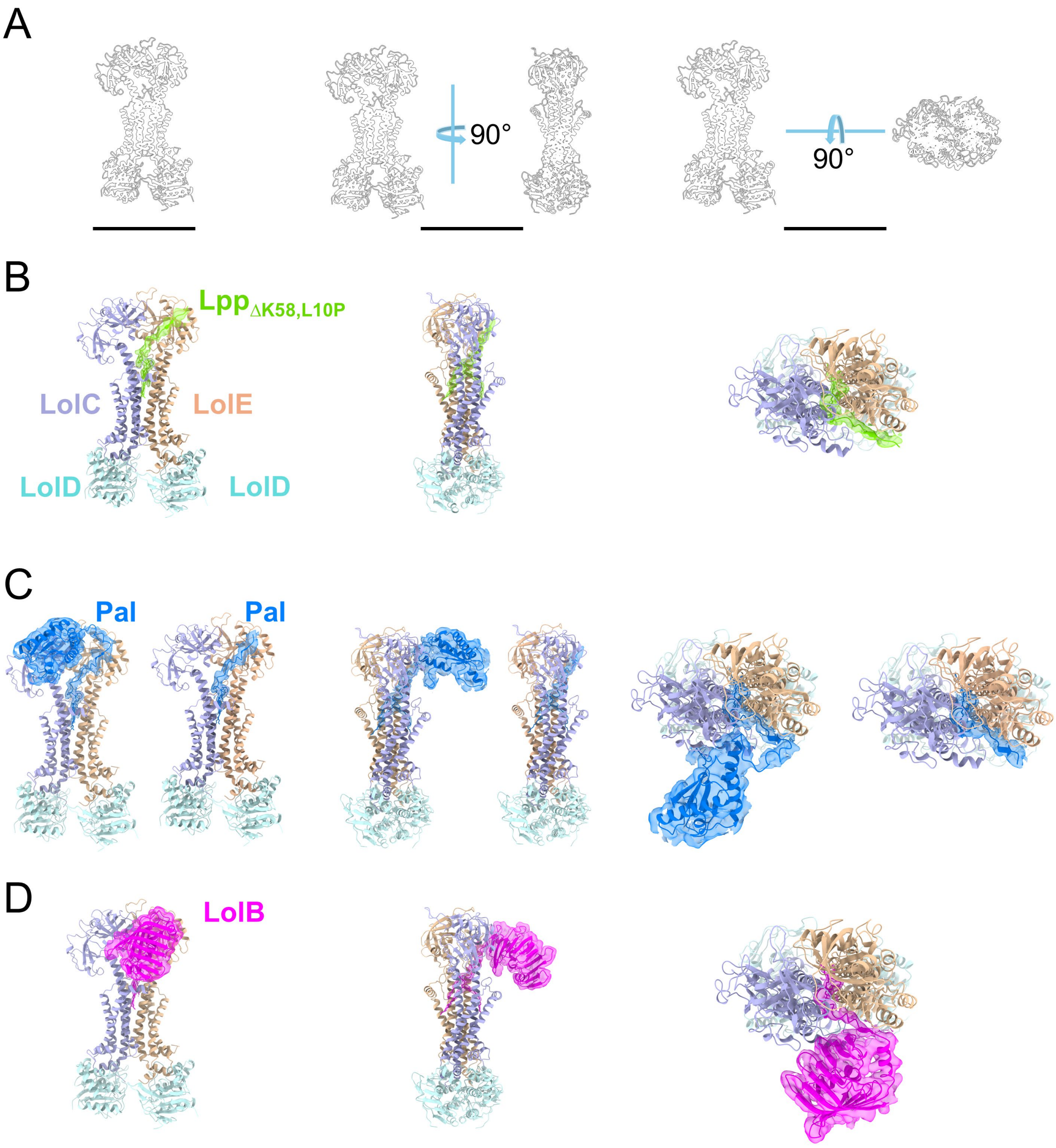

Fig. S2. Ribbon model views of cryoEM structures from Fig. 1 with substrate map zones. (A) Orientation matrix for columns in rows (B-D) below. (B) LolCDE complexed with $Lpp_{\Delta K58,L10P}$ (C) LolCDE complexed with Pal. (D) LolCDE complexed with LolB.

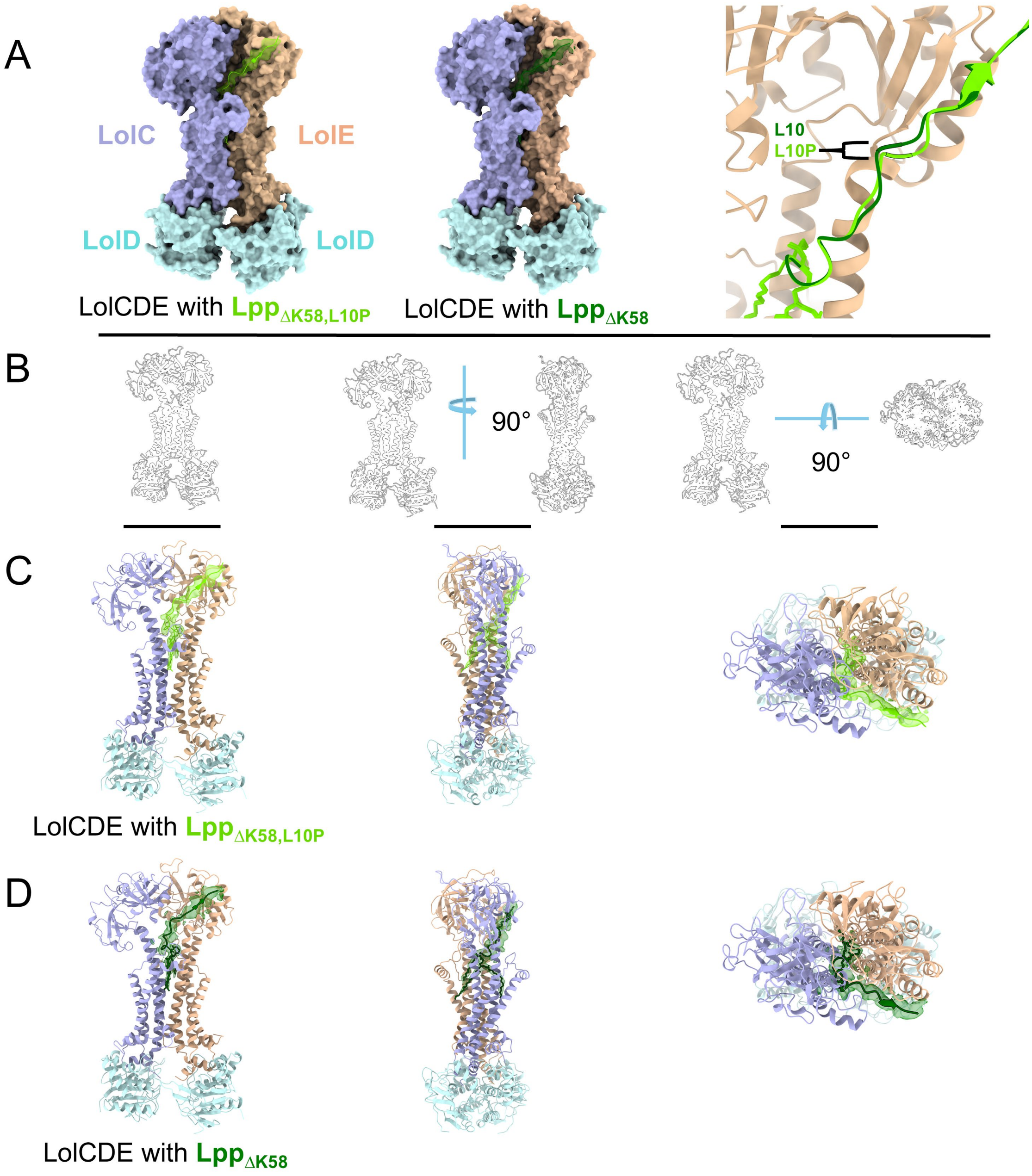

Fig. S3. Ribbon model views of Lpp$_{\Delta K58,L10P}$ (2.9Å) and Lpp$_{\Delta K58}$ cryoEM (3.0Å) structures with substrate map zones. (A) Space filled models with lipoprotein ribbon cartoon and semi-transparent lipoprotein map zone overlays for Lpp$_{\Delta K58,L10P}$ and Lpp$_{\Delta K58}$. (*left*) Superposition of linkers from the LppΔK58,L10P and LppΔK58 complexes showing slight trajectory deviation maximized about linker residue 10 (*right*). (B) Orientation matrix for columns in rows (C-D) below. (C) LolCDE complexed with Lpp$_{\Delta K58,L10P}$. (D) LolCDE complexed with Lpp$_{\Delta K58}$.

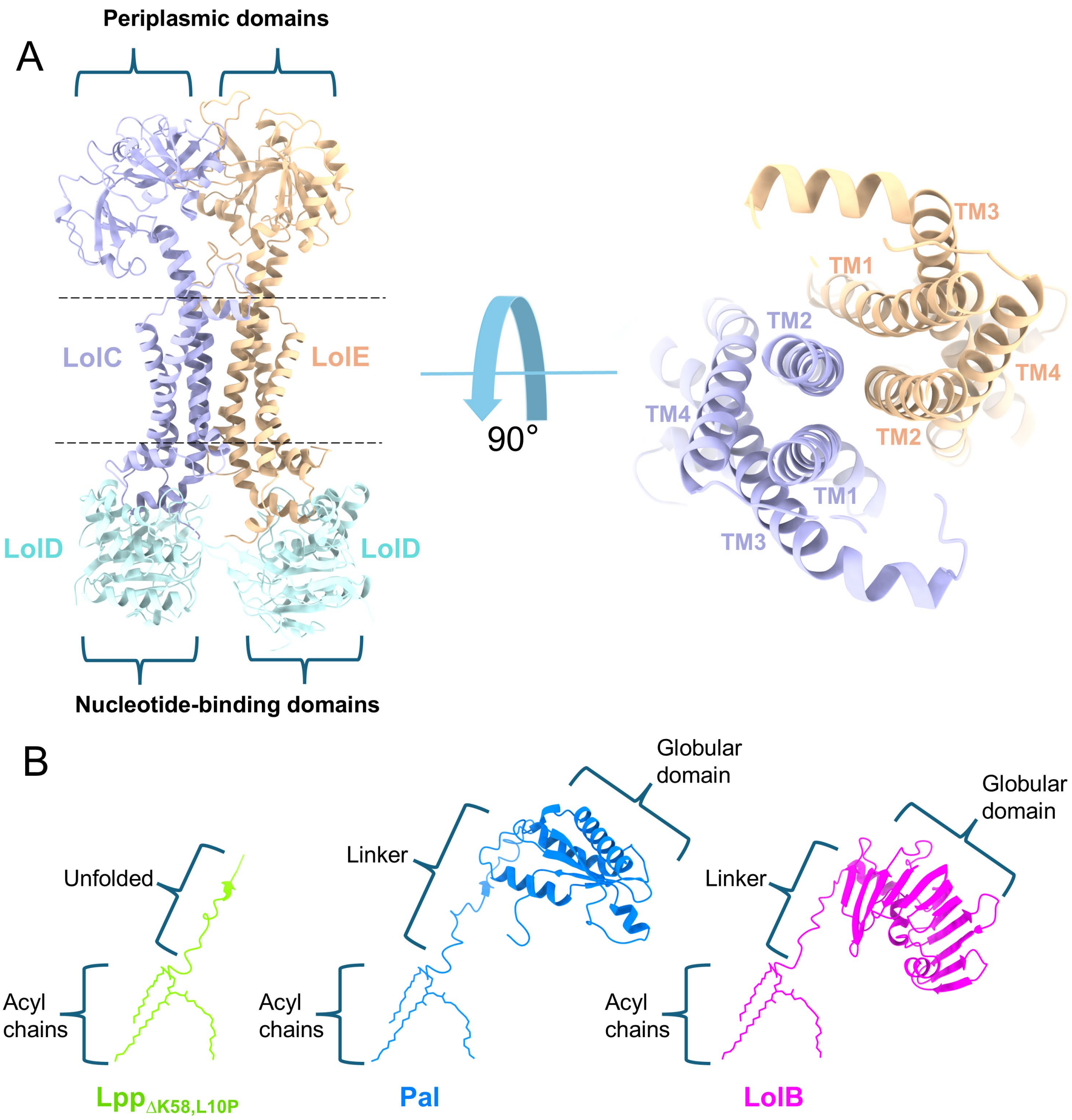

Fig. S4. Ribbon model topology of LolCDE and ribbon models of acylated lipoproteins. (A) Ribbon model of LolCDE showing the locations of the periplasmic and nucleotide-binding domains. (*right*) 90° rotation of the area denoted by dotted lines in (A) viewed down from the periplasm towards the inner membrane with the transmembrane helices of LolCDE labelled. (B) Ribbon views of $Lpp_{\Delta K58,L10P}$, Pal, and LolB with the linker or unfolded regions bounding the SβS, acyl chains, and globular domains indicated.

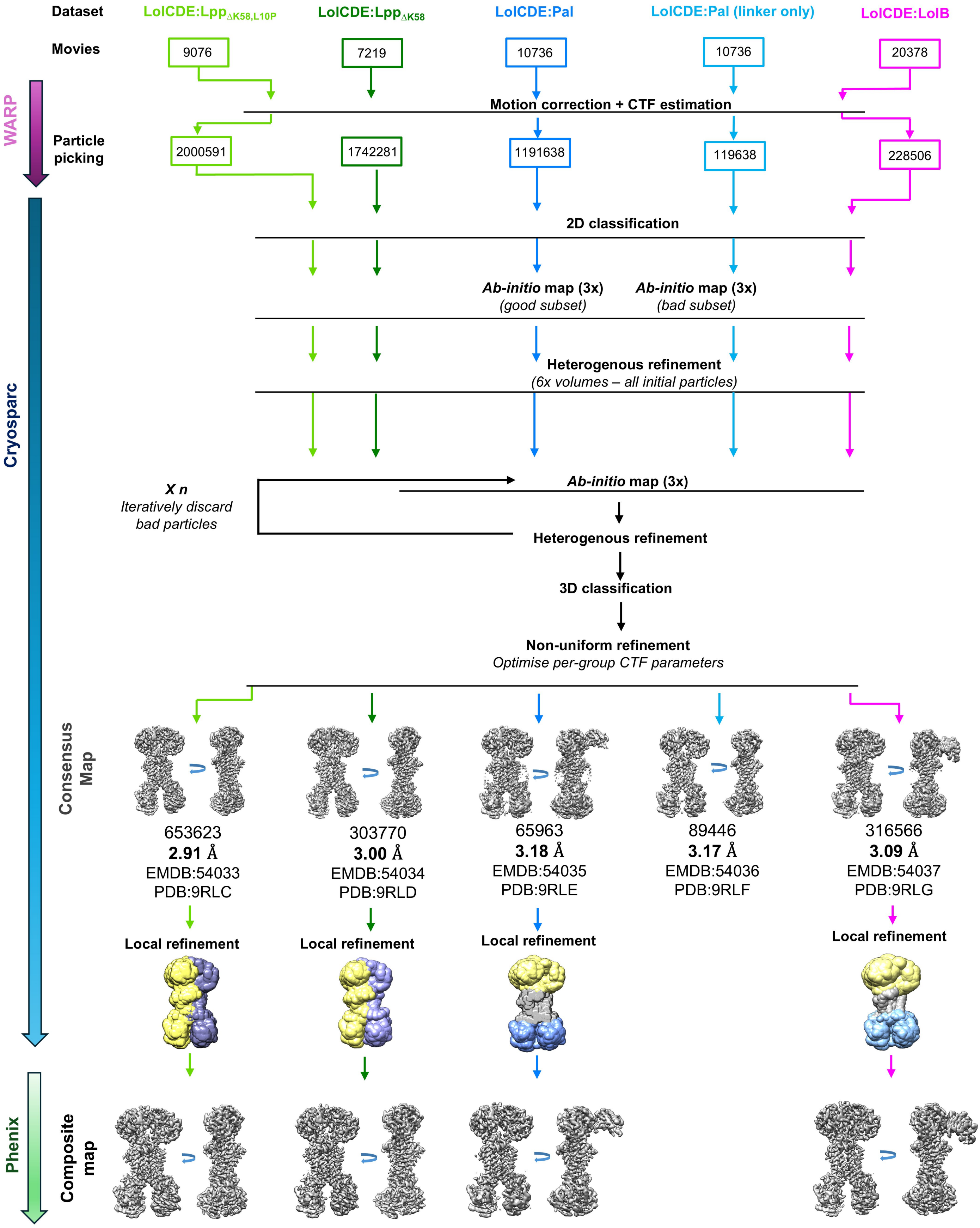

Fig S5. Image processing workflow for the LolCDE substrate complexes

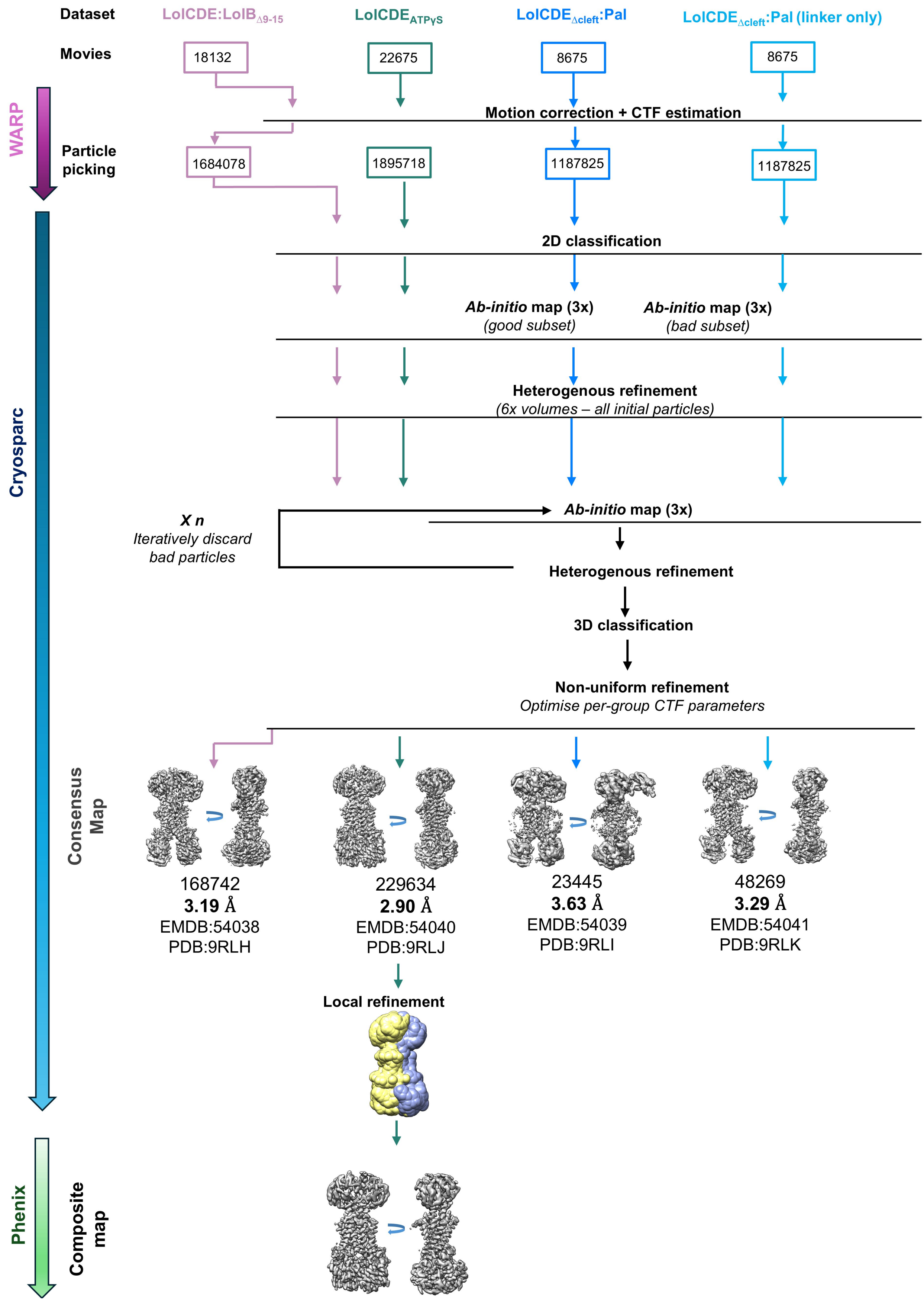

Fig S5 cont. Image processing workflow for the LolCDE substrate complexes

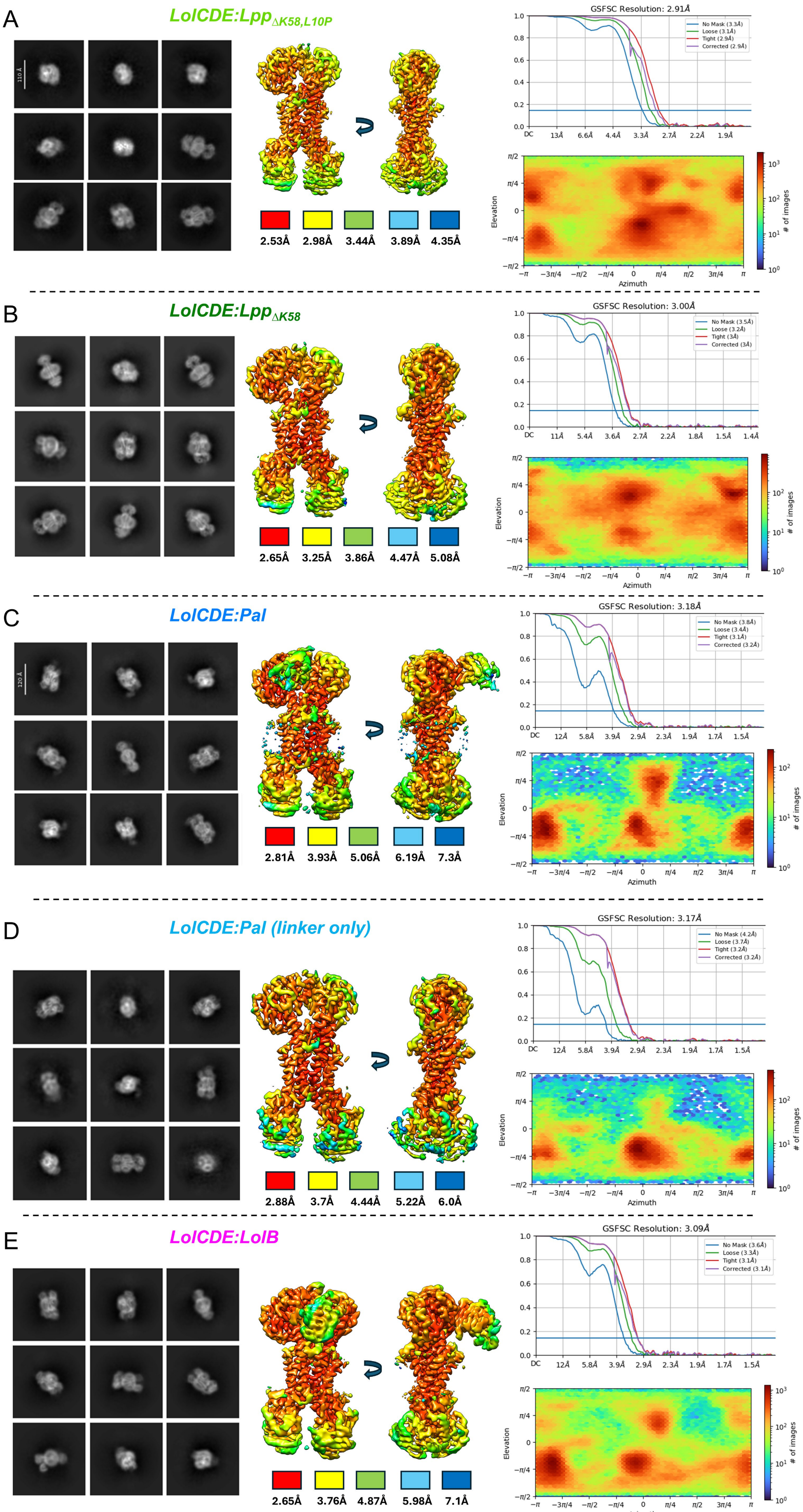
A
LolCDE:LppΔK58,L10P
2.53Å 2.98Å 3.44Å 3.89Å 4.35Å
GSFSC Resolution: 2.91Å
No Mask (3.3Å)
Loose (3.1Å)
Tight (2.9Å)
Corrected (2.9Å)
Elevation
Azimuth
# of images
B
LolCDE:LppΔK58
2.65Å 3.25Å 3.86Å 4.47Å 5.08Å
GSFSC Resolution: 3.00Å
No Mask (3.5Å)
Loose (3.2Å)
Tight (3Å)
Corrected (3Å)
C
LolCDE:Pal
2.81Å 3.93Å 5.06Å 6.19Å 7.3Å
GSFSC Resolution: 3.18Å
No Mask (3.8Å)
Loose (3.4Å)
Tight (3.1Å)
Corrected (3.2Å)
D
LolCDE:Pal (linker only)
2.88Å 3.7Å 4.44Å 5.22Å 6.0Å
GSFSC Resolution: 3.17Å
No Mask (4.2Å)
Loose (3.7Å)
Tight (3.2Å)
Corrected (3.2Å)
E
LolCDE:LolB
2.65Å 3.76Å 4.87Å 5.98Å 7.1Å
GSFSC Resolution: 3.09Å
No Mask (3.6Å)
Loose (3.3Å)
Tight (3.1Å)
Corrected (3.1Å)

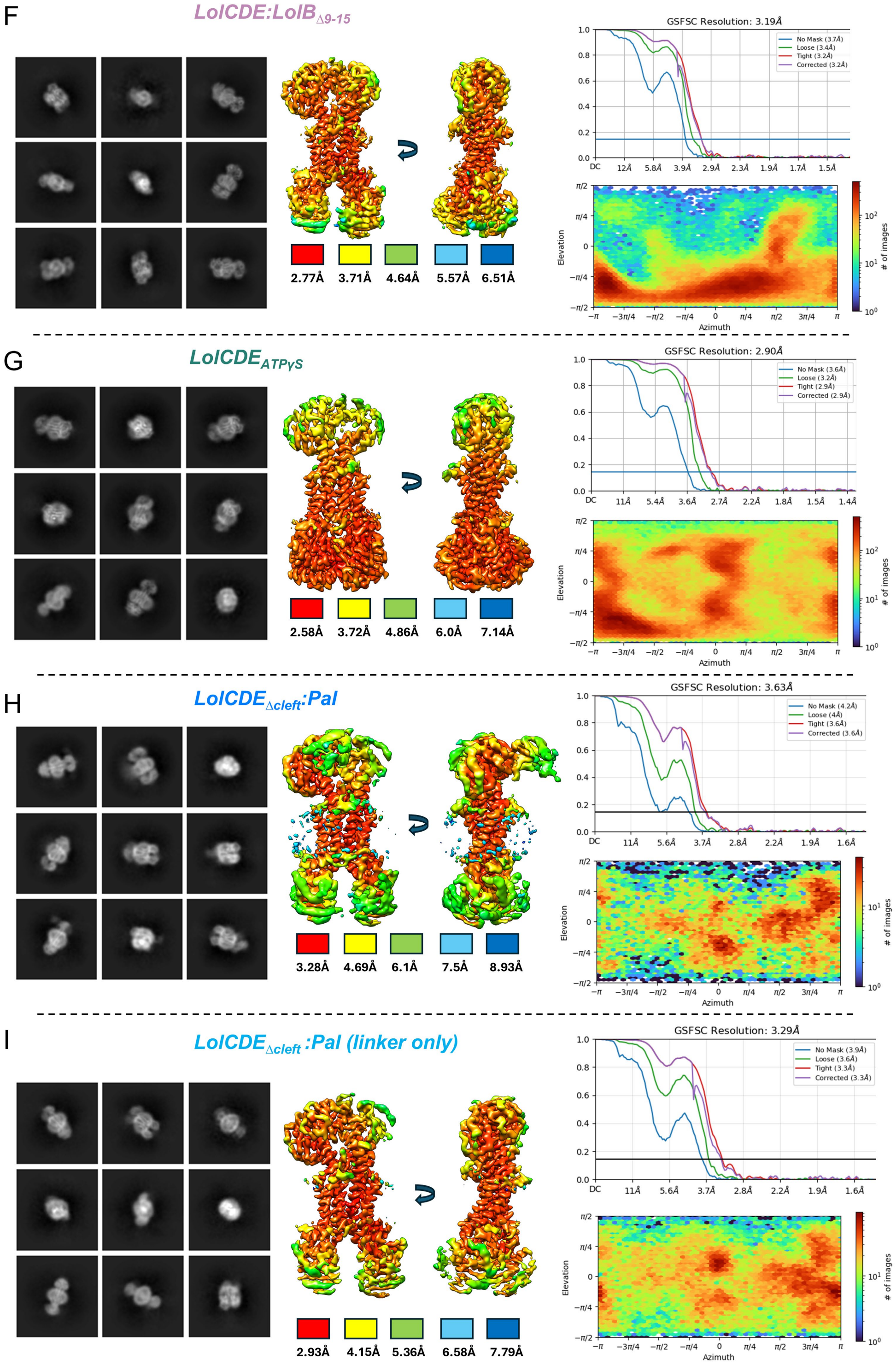

Fig. S6. Cryo-EM analysis of the LolCDE complexes. (A) LolCDE:Lpp$_{\Delta K58.L10P}$, (B) LolCDE:Lpp$_{\Delta K58}$, (C) LolCDE:Pal, (D) LolCDE:Pal (linker only), (E) LolCDE:LolB, (F) LolCDE:LolB$_{\Delta 9\text{-}15}$, (G) LolCDE$_{ATP\gamma S}$, (H) LolCDE$_{\Delta cleft}$:Pal, (I) LolCDE$_{\Delta cleft}$:Pal (linker only). For each structure, representative 2D class averages generated in cryoSPARC (*left*). Maps coloured by local resolution calculated in CryoSPARC (*centre*). Fourier Shell Correlation (FSC) plots for the refinements shown. Resolution is indicated at the FSC=0.143 cut-off *(right, upper)*. Angular distribution plot of the final particle set after consensus refinement (*right, lower*).

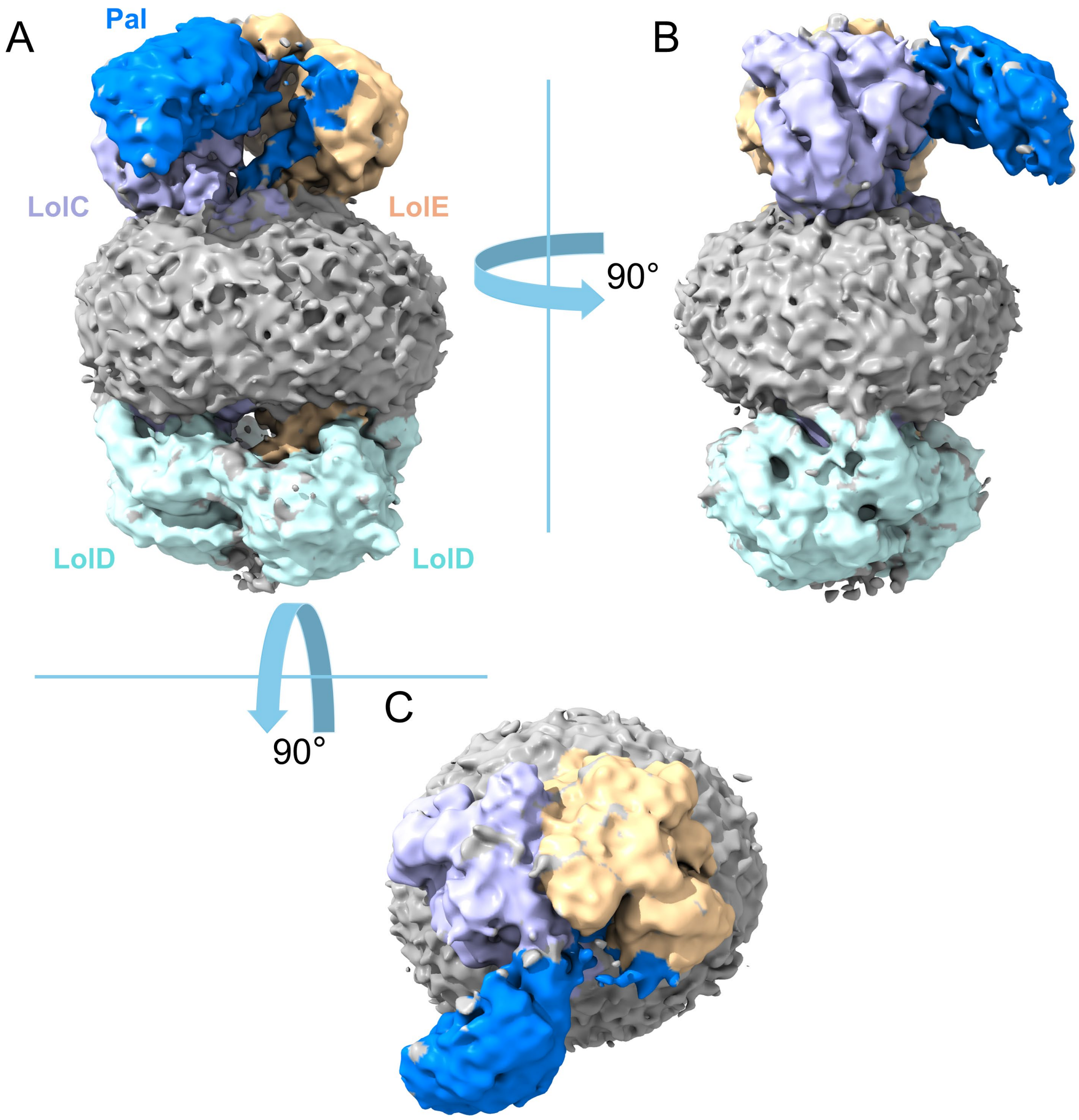

Fig. S7. Map of untagged Pal complexed with LolCDE at 3.77Å. Surface overlay coloured to aid viewing from our 3.18Å LolCDE:Pal model and coloured as same. (A) Origin view. (B-C) Rotational views from (A) as indicated.

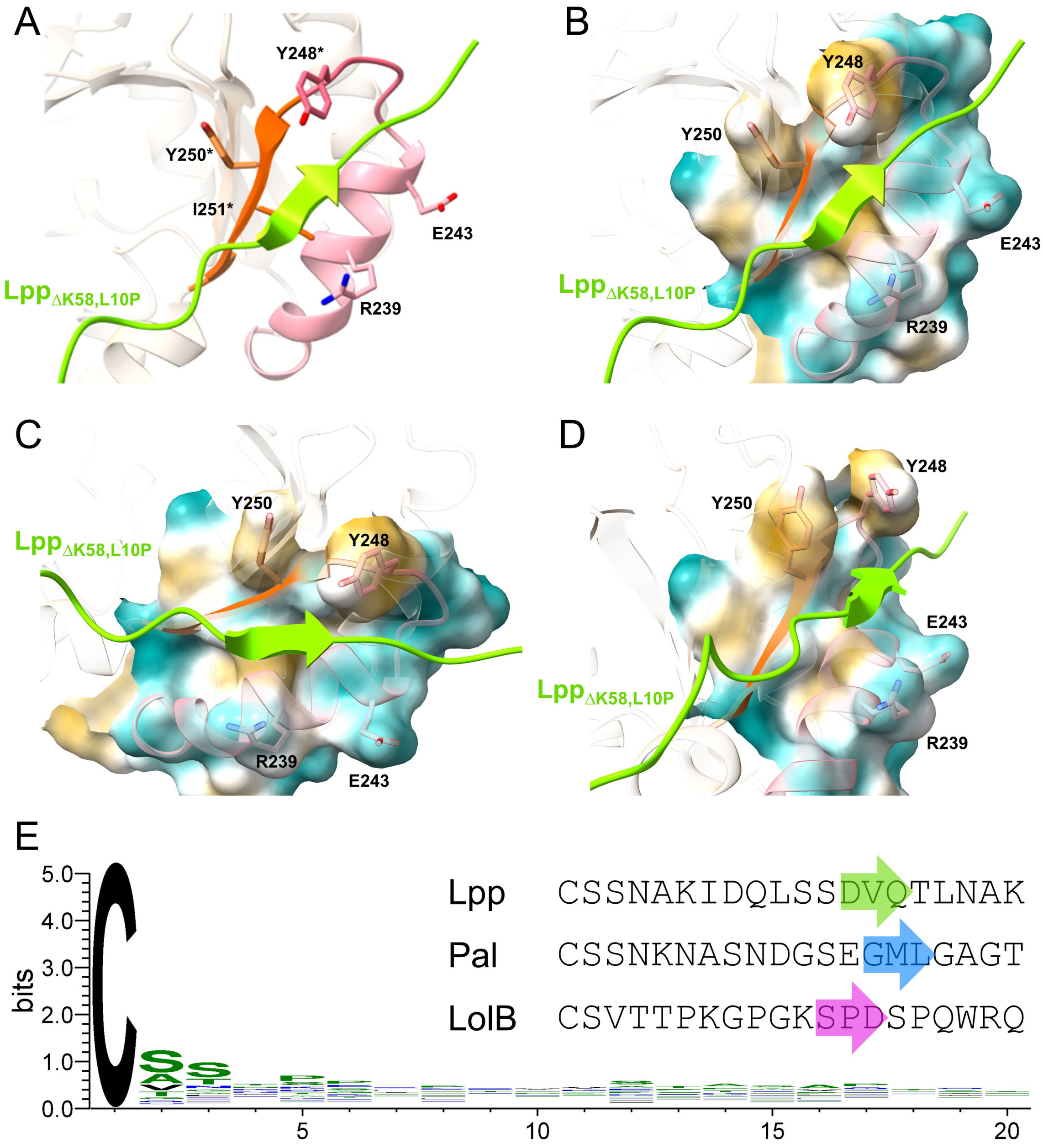

Fig. S8. Lipoprotein interaction with the LolE cleft. (A) Pathway of $Lpp_{\Delta K58,L10P}$ through the LolE cleft, coloured as in Fig. 2B, with $Lpp_{\Delta K58,L10P}$ in green. Residues that formed crosslinks with BamE when substituted with p-benzoyl-L-phenylalanine (1) are indicated with asterisks. (B-D) Views of the cleft surface shown in transparent lipophilicity map of LolE coloured from cyan (most hydrophilic), to gold (most lipophilic). (E) Conservation plot of the N-terminal 20 residues of mature, experimentally confirmed outer membrane *E. coli* lipoproteins identified by Sueki *et al* (2). Plot generated in Weblogo (3). Sequences of Lpp, Pal, and LolB with the position of the substrate β-strand indicated by an arrow are shown inset.

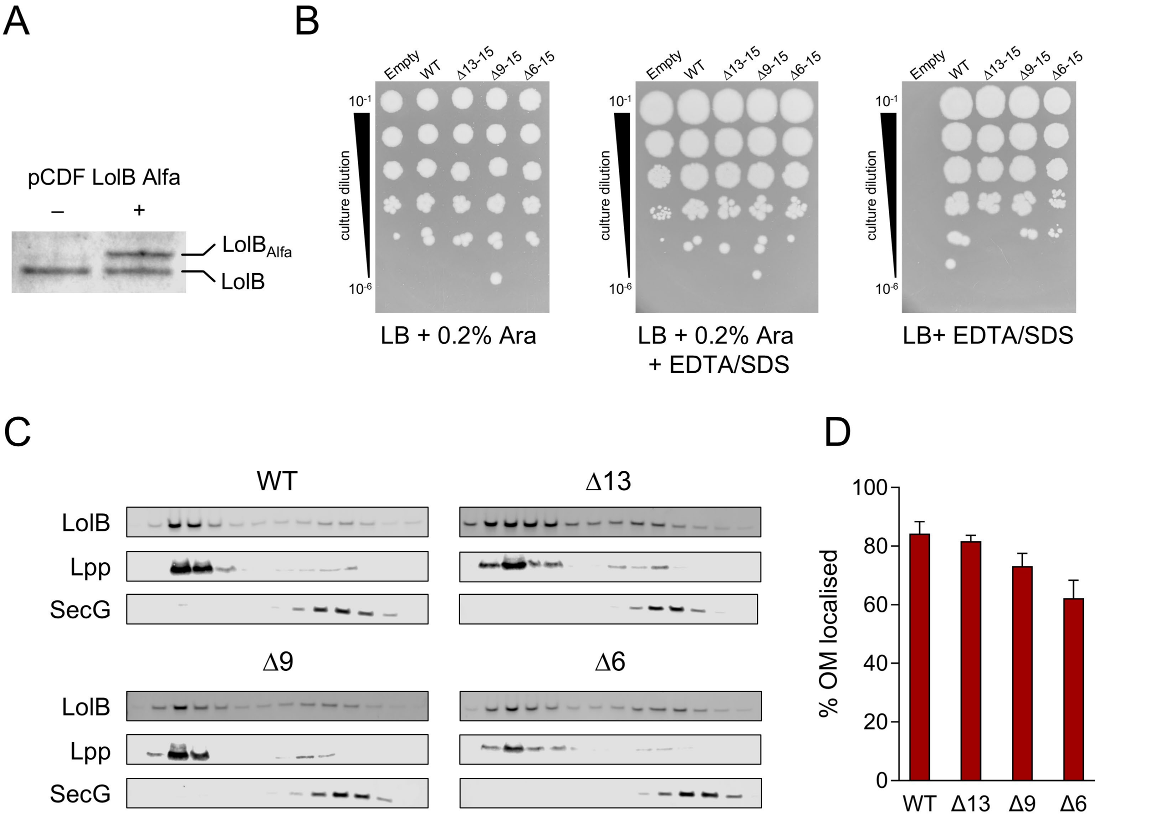

Fig. S9. ***In vivo* analysis of truncations in the LolB linker**. (A) Whole cell immunoblot of BW25113 cells bearing empty vector (-) or pCDF $LolB_{Alfa}$ (+) showing relative expression of plasmid-borne $LolB_{Alfa}$ with chromosomally encoded LolB from the native *lolB* promoter. (B) Serial dilutions of the conditional *lolB* knockout strain, BW65, cells bearing empty vector or expressing plasmid-borne wild-type LolB or indicated $LolB_{\Delta X\text{-}Y}$ deletion variants grown on LB plates with the indicated additions. 0.2% arabinose induces expression of the chromosomal wild-type *lolB* gene while 0.5 mM EDTA/0.5% SDS induces membrane stress. (C) Illustrative immunoblots of samples from fractionated membranes of strain BW65 bearing pCDF $LolB_{Alfa}$ or the indicated deletion variant. SecG and Lpp were used as markers for the inner and outer membrane respectively. (D) Quantification of the proportion of wild-type LolB or indicated deletion variant that localised to the outer membrane. Results are the mean of three independent fractionations.

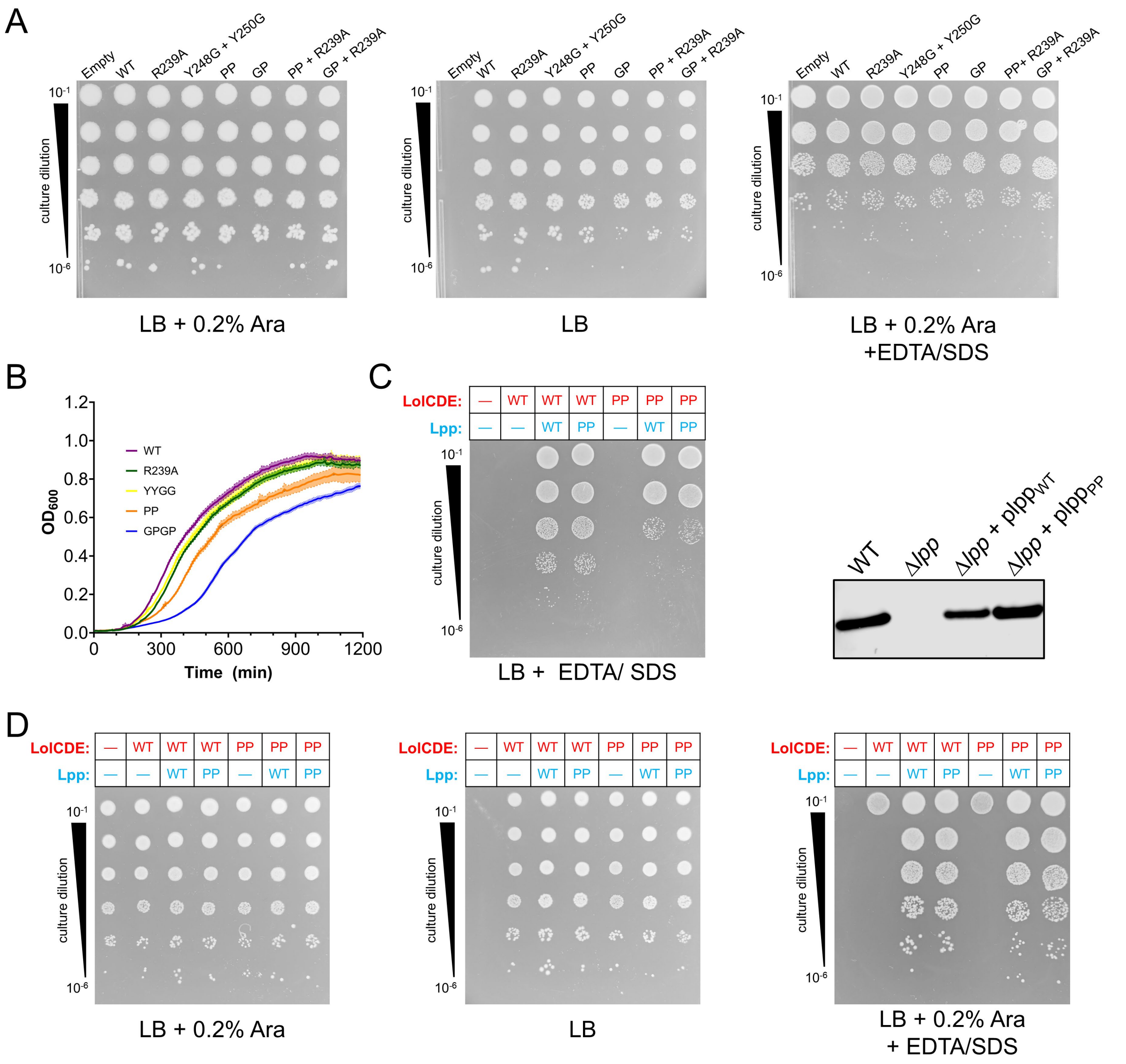

Fig S10. *In vivo* analysis of mutations in the LolE cleft (A) Serial dilutions of a conditional *lolCDE* knockout *E. coli* strain (HD200313) carrying either plasmid-borne wild-type *lolCDE* or variant plated on LB medium with the indicated additions. 0.2% arabinose induces expression of the chromosomal wild-type *lolB* gene while 0.5 mM EDTA/0.5% SDS induces membrane stress. (B) Growth of *lolCDE* knockout *E. coli* strain (HD200313) carrying plasmid-borne wild-type *lolCDE* or indicated variant in liquid culture (LB) in the absence of stress. Growth was followed at OD600 with curves depicting the mean ± SD for three independent cultures. (C) Serial dilutions of HD200313 Δ*lpp* bearing empty vector (–), plasmid-borne wild-type *lolCDE* (WT) or *lolCDE* where *lolE* contains prolines at positions 249 and 251 (PP) and wild-type *lpp* (WT) or D13P+Q15P (PP) variants (*left*). Immunoblot showing expression of native *lpp* in HD200313 (WT) or plasmid-borne *lpp* ($pLpp_{WT}$) or D13P+Q15P variant ($pLpp_{PP}$) in HD200313 Δ*lpp* (*right*). (D) Control plates for the experiment shown in (C) showing growth of HD200313 Δ*lpp* bearing empty vector or *lolCDE* or *lpp* constructs labelled as in (C). Cells were grown on LB agar with additions as indicated. 0.2% arabinose induces expression of the chromosomal wild-type *lolCDE* gene while 0.5 mM EDTA/0.5% SDS induce membrane stress.

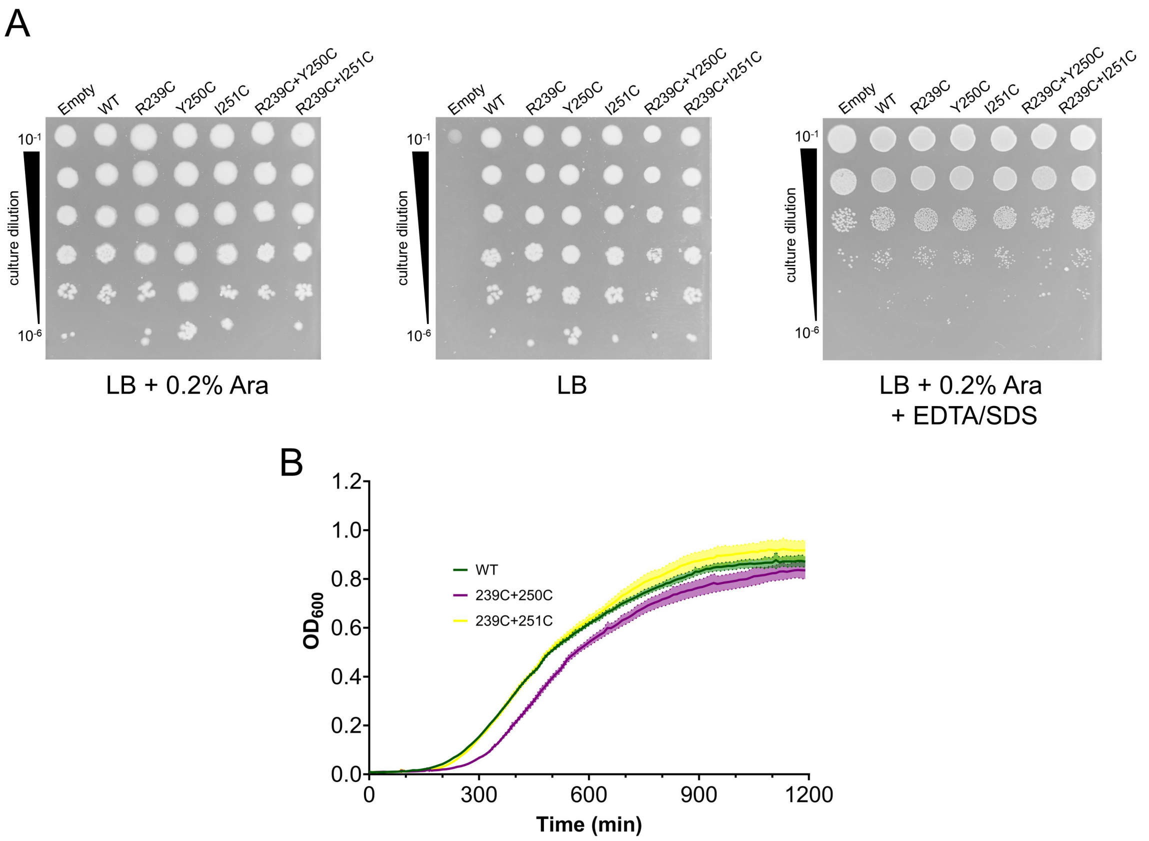

Fig S11. *In vivo* analysis of cells expressing LolE cysteine variants. (A) Serial dilutions of a conditional *lolCDE* knockout *E. coli* strain (HD200313) carrying either plasmid-borne wild-type *lolCDE* or indicated *lolE* cysteine variants on LB agar containing the indicated additions, 0.2% arabinose induces expression of the chromosomal wild-type *lolCDE* gene while 0.5 mM EDTA/0.5% SDS induce cell envelope stress. (B) Growth of *lolCDE* knockout *E. coli* strain (HD200313) carrying plasmid-borne wild-type *lolCDE* or indicated cysteine variant in liquid culture (LB-medium) in the absence of stress. Growth was followed at OD600 with curves depicting the mean ± SD for three independent cultures.

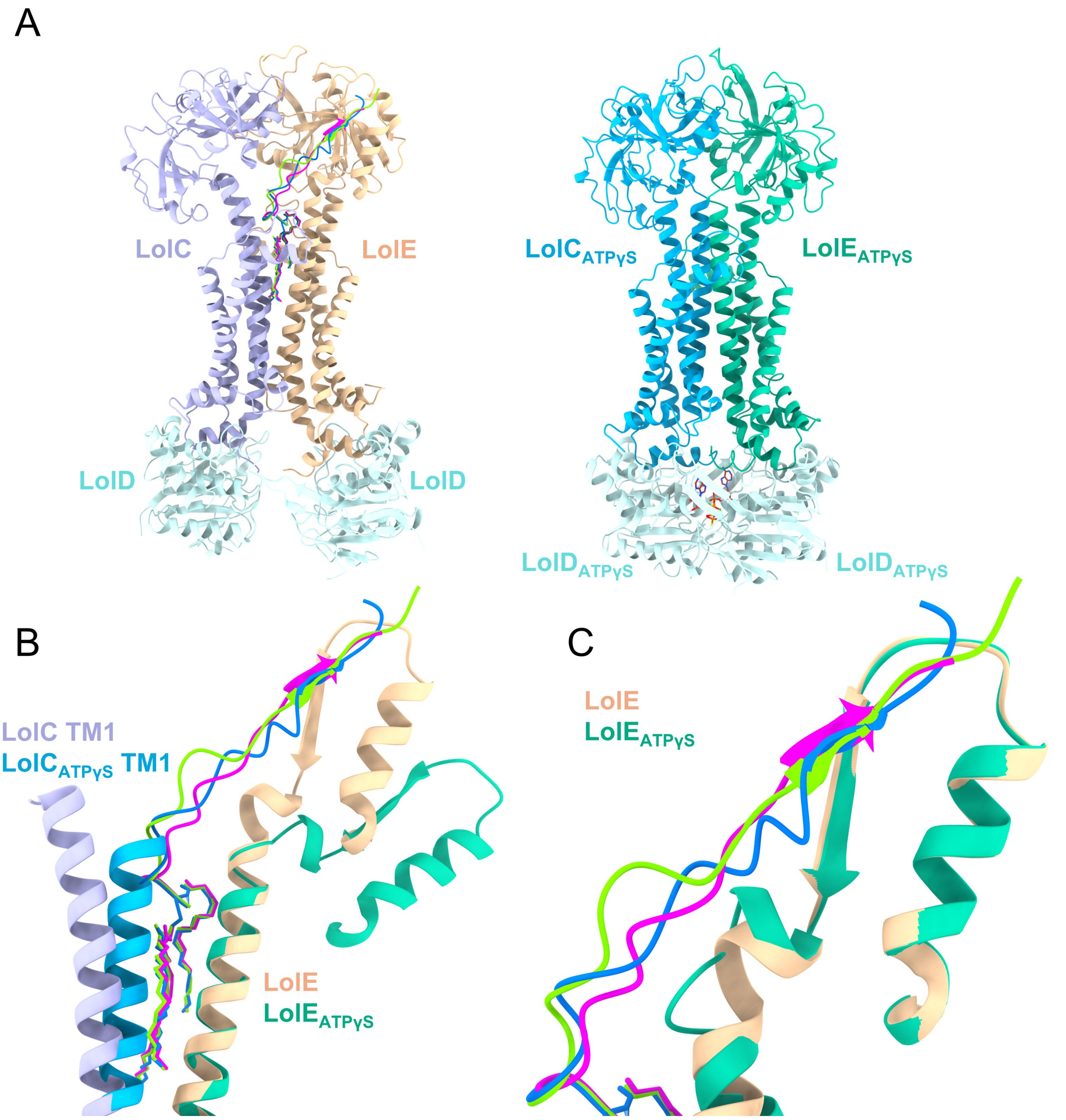

Fig S12. Alignment of LolCDE lipoprotein-bound models with LolCDE$_{ATP\gamma S}$. (A) Ribbon models of LolCDE:lipoprotein (LolCDE with a segment of the Lpp$_{\Delta K58,L10P}$ (light green), Pal (blue), and LolB (pink) linkers overlaid) (*left*), and LolCDE$_{ATP\gamma S}$ (*right*). (B) LolCDE$_{ATP\gamma S}$ aligned to LolCDE:lipoprotein using LolE TM2 (LolE residues 255-308) of both. (C) LolCDE$_{ATP\gamma S}$ aligned to LolCDE:lipoprotein using the LolE cleft (LolE residues 231-253) of both.

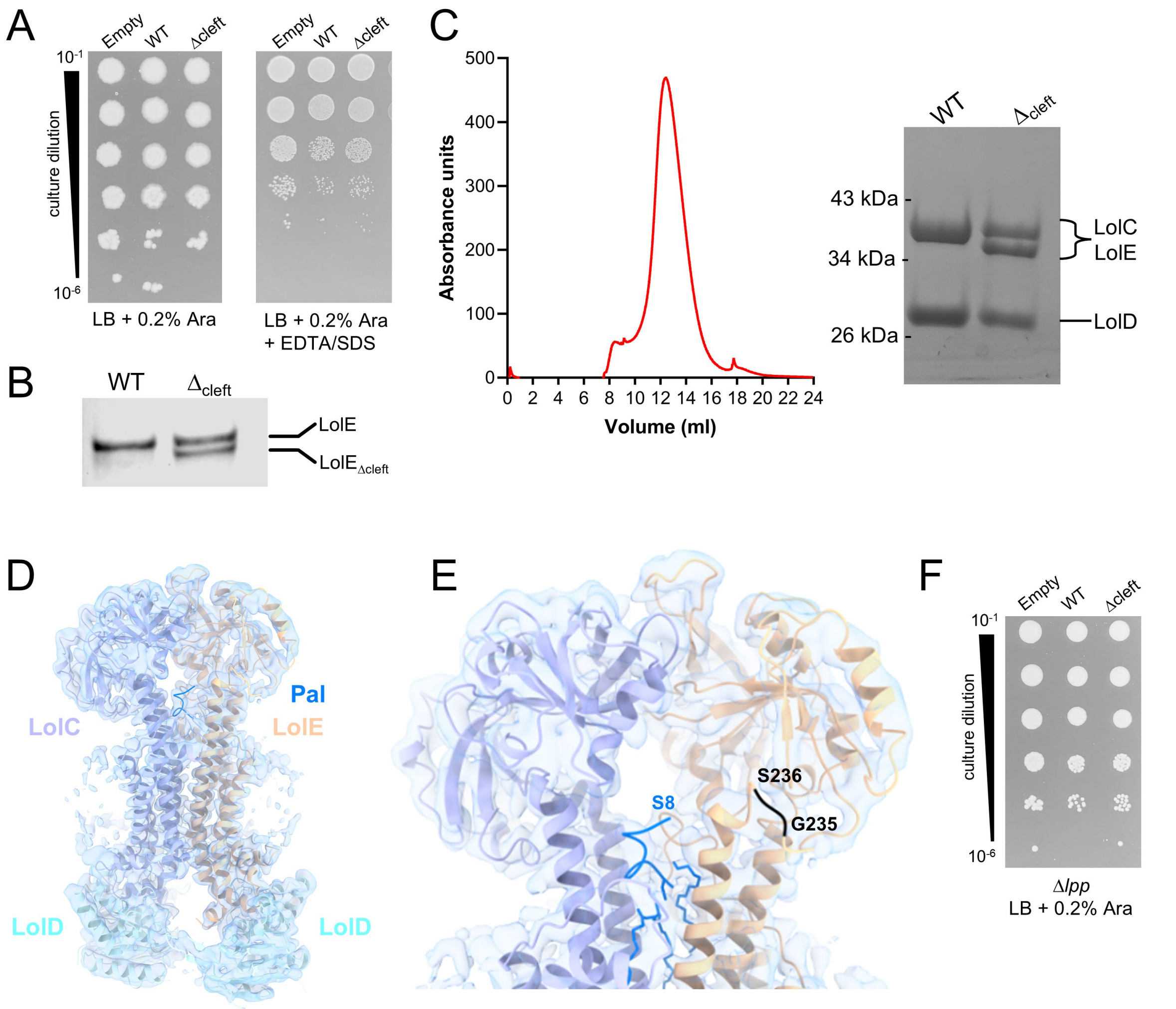

Fig. S13. *In vivo* and structural characterisation of the LolCDE$_{\Delta cleft}$ variant (A) Control plates for Fig. 6B. Serial dilutions of a conditional *lolCDE* knockout *E. coli* strain (HD200313) carrying either plasmid-borne wild-type *lolCDE* or *lolCDE$_{\Delta cleft}$* on LB agar containing the indicated additions, 0.2% arabinose induces expression of the chromosomal wild-type *lolCDE* gene while 0.5 mM EDTA/0.5% SDS induces cell envelope stress. (B) Immunoblot of LolE from complexes purified from cells grown in (A) showing expression of wild-type LolE and LolEΔ$_{cleft}$. (C) Size exclusion chromatography trace of purified LolCDE$_{\Delta cleft}$:Pal complex (*left*) with SDS-PAGE showing purified wild-type LolCDE:Pal versus LolCDE$_{\Delta cleft}$:Pal (*right*). (D) Ribbon model of LolCDE$_{\Delta cleft}$:Pal state with the Pal globular domain unresolved at 3.29Å, map overlaid in light blue. (E) Close-in view of (D). The GS linker that replaces the LolE cleft (*black*) and the resolved Pal linker (*blue)* are indicated. (F) Control plates for Fig. 6F. Serial dilutions of HD200313 Δ*lpp* carrying empty vector or either plasmid-borne wild-type *lolCDE* or *lolCDE*$_{\Delta cleft}$ as indicated. Cells were grown on 0.2% arabinose to induce expression of chromosomally encoded *lolCDE*.

Table S1. Data collection and refinement statistics

	LolCDE:Lpp$_{\Delta K58,L10P}$	LolCDE:Lpp$_{\Delta K58}$	LolCDE:Pal Pal domain present	LolCDE:Pal Pal linker only	LolCDE:LolB
	PDB:9RLC EMDB:54033	PDB:9RLD EMDB:54034	PDB:9RLE EMDB:54035	PDB:9RLF EMDB:54036	PDB:9RLG EMDB:54037
**Data collection and processing**					
Detector	Gatan K3	Gatan K3	Gatan K3	Gatan K3	Gatan K3
Magnification	105K	130k	130k	130k	130k
Energy filter slit width (eV)	20	20	20	20	20
Voltage (kV)	300	300	300	300	300
Flux on detector (e/pix/sec)	18.44	22.01	20.96	20.96	17.77
Electron exposure on sample (e–/Å^2)	47.37	51.77	51.29	51.29	55.59
Target defocus range (μm)	1.0-2.4	0.8-2.2	0.8-2.2	0.8-2.2	1.0-2.6
Calibrated pixel size (Å)	0.830	0.652	0.652	0.652	0.652
Symmetry imposed	C1	C1	C1	C1	C1
Initial particle images (no.)	2000591	1742281	1191638	119638	2228506
Final particle images (no.)	653623	303770	65963	89446	316566
**Refinement**					
Map resolution at FSC=0.143 (Å)*	2.91	3.00	3.18	3.17	3.09
Model composition					
Non-hydrogen atoms	9870	9844	10952	9840	11216
Protein residues	1283	1280	1423	1281	1449
Nucleotides	0	0	0	0	0
CC(mask)	84.6%	84.9%	83.8%	84.4%	82.3%
B factor (Å^2)					
Protein	130.7	125.6	160.6	191.6	156.1
Ligand	110.9	112.9	139.9	177.6	141.4
R.m.s deviations					
Bond lengths (Å)	0.003	0.003	0.002	0.003	0.003
Bond angles (°)	0.502	0.555	0.537	0.554	0.554
Validation					
Molprobity score	1.62	1.60	1.63	1.61	1.52
Clashscore	6.11	6.47	6.61	6.43	5.96
Poor rotamers (%)	0	0	0	0	0
Ramachandran plot					
Favored (%)	95.83	96.38	96.18	96.76	96.87
Allowed (%)	4.17	3.62	3.82	3.78	3.13
Disallowed (%)	0.00	0.00	0.00	0.00	0.00

	$LolCDE:LolB_{\Delta 9\text{-}15}$	$LolCDE_{ATP\gamma S}$	$LolCDE_{\Delta cleft}$:Pal	$LolCDE_{\Delta cleft}$:Pal	$LolCDE_{CC}$:Pal	$LolCDE_{CC}$:Pal
			Pal domain present	Pal linker only	open state	closed state
	PDB:9RLH	PDB:9RLJ	PDB:9RLI	PDB:9RLK		
	EMDB:54038	EMDB:54040	EMDB:54039	EMDB:54041	EMDB:54479	EMDB:54478
**Data collection and processing**						
Detector	Gatan K3	Gatan K3	Falcon 4i	Falcon 4i	Falcon 4i	Falcon 4i
Magnification	130k	130k	165k	165K	165K	165K
Energy filter slit width (eV)	20	20	10	10	10	10
Voltage (kV)	300	300	300	300	300	300
Flux on detector (e/pix/sec)	21.58	18.56	6.44	6.44	6.64	6.35
Electron exposure on sample (e–/Å^2)	52.79	52.39	53.20	53.20	54.85	52.45
Target defocus range (μm)	0.8-2.2	0.8-2.2	0.6-1.8	0.6-1.8	0.8-2.2	0.8-2.0
Calibrated pixel size (Å)	0.652	0.652	0.729	0.729	0.729	0.729
Symmetry imposed	C1	C1	C1	C1	C1	C1
Initial particle images (no.)	1684078	1895718	1187825	1187825	1530343	678934
Final particle images (no.)	168742	229634	23445	48269	102654	92720
**Refinement**						
Map resolution at FSC=0.143 (Å)*	3.19	2.90	3.63	3.29	3.48	3.26
Model composition						
Non-hydrogen atoms	9869	9775	10673	9647		
Protein residues	1281	1268	1384	1256		
Nucleotides	0	2	0	0		
CC(mask)	83.8%	80.3%	82.1%	83.8%		
B factor (Å^2)						
Protein	132.0	180.1	193.2	167.8		
Ligand	112.3	132.3	156.8	132.0		
R.m.s deviations						
Bond lengths (Å)	0.004	0.006	0.003	0.003		
Bond angles (°)	0.603	0.559	0.625	0.659		
Validation						
Molprobity score	1.61	1.80	1.71	1.71		
Clashscore	6.31	7.22	7.80	6.86		
Poor rotamers (%)	0	0	0	0		
Ramachandran plot						
Favored (%)	96.22	94.05	95.84	95.50		
Allowed (%)	3.78	5.95	4.16	4.50		
Disallowed (%)	0.00	0.00	0.00	0.00		

Validation metrics and B-factor statistics reported are from PHENIX (4) and Molprobity (5, 6)

Table S2. Structural comparison of LolCE proteins in the open and closed states of LolCDE.

	RMSD Cα Å (max)	9GRC (1)	9RLD (this work)	7V8L (7)
Open forms	Residues > 3.8Å	LolC/LolE	LolC/LolE	LolC/LolE
	9GRC (1)		1.54 (7.36)	2.10 (10.81)
	9RLJ (this work).	23/17		1.52 (8.64)
	7V8I (7)	39/21	16/15	

	RMSD Cα Å (max)	9GVK (1)	9RLJ (this work)	7V8I (7)
Closed forms	Residues > 3.8Å	LolC/LolE	LolC/LolE	LolC/LolE
	9GRC (1)		3.23 (19.3)	3.94 (15.6)
	9RLJ (this work).	33/59		3.60 (14.4)
	7V8I (7)	61/187	38/165	

RMSD values (Å), with maximum displacement indicated in brackets between the indicated structures (blue shaded boxes). Number of residues displaced by >3.8 Å for LolC/LolE chains (orange shaded boxes).

Table S3. List of primers for PCR amplification.

Primer	Description	Sequence (5' to 3')
P1	*lpp* KO_F	AAACTTTGTGTAATACTTGTAACGCTACATGGAGATTAACTCAAT CTAGAGGGTATTAATAATGATTCCGGGGATCCGTCGACC
P2	*lpp* KO_R	AAAAAATGGCGCACAATGTGCGCCATTTTTCACTTCACAGGTAC TATTACTTGCGGTATTTAGTTGTAGGCTGGAGCTGCTTCG
P3	*lpp* KO screen_F	AGCGTTCGATGCTTCTTTGAGC
P4	*lpp* KO screen_R	AGCAATGCGCCTTCAGTAGAGTC
P5	BAD18_lolB_F	GCGCGCTAGCAACTGCAACGTATCTCAAGGACTTGTCATCACTA TGCCCCTGCCCGATTTTCGTCTTATC
P6	BAD18_lolB_R	GCGCAAGCTTTTATTTCACTATCCAGTTATCCATTTTTAAC
P7	araBAD_lolB_F	GCGCATCGATGCATAATGTGCCTGTCAAATGGACG
P8	araBAD_lolB_R	GCGCGAGCTCTAGAAACGCAAAAAGGCCATCCGTCAGGATG
P9	lolB_KO_F	GGACGGTAACGCTAGCATTAAGGGTTATAACTGCAACGTATCTC AAGGACTTGTCATCACTATGATTCCGGGGATCCGTCGACC
P10	lolB_KO_R	ATAAAAACAGATTAAGTTTGCCGGAGAGGGCCACTGTGTCCGC ATTATTTCACTATCCAGTTATCTGTAGGCTGGAGCTGCTTCG
P11	lolB_screen_F	CTTTTGGAGTAATTGCCGAAAGC
P12	lolB_screen_R	CCGTAATCAAGAAACTGAAACAGC
P13	*lpp* Δ78 strep_F	GTTTAACTTTAATAAGGAGATATACCATGAAAGCTACTAAACTG GTACTG
P14	Twin-strep_R	GTTTCTTTACCAGACTCGAGGGTACTTATTTCTCGAACTGCGGGT GGCTC
P15	*pal* strep_F	GTTTAACTTTAATAAGGAGATATACCATGCAACTGAACAAAGTG CTGAAAGGGC
P16	*lolB* strep_F	GTTTAACTTTAATAAGGAGATATACCATGCCACTGCCAGATTTTC GTC
P17	*lpp* untagged_F	ACGCTCTCCCTTATGCGACTCCTGCTTGATGGCTAGCTCAGTCCT AGGTAC
P18	*lpp* untagged_R	GTTTCTTTACCAGACTCGAGGGTACACAAAAAAAATGGCGCACA ATGTGCGC
P19	*lolB* Alfa-tag_F	GTTTAACTTTAATAAGGAGATATACCATGCCACTGCCAGATTTTC GTC
P20	*lolB* Alfa-tag_R	GTTTCTTTACCAGACTCGAGGGTACTTATTCGGTCAGACGGCGAC GC
P21	*lpp* L10P_F	CGCTAAAATCGATCAGCCGTCTTCTGACGTTCAG
P22	*lpp* L10P_R	CTGAACGTCAGAAGACGGCTGATCGATTTTAGCG
P23	*lpp* D13P Q15P_F	CGATCAGCTGTCTTCTCCGGTTCCGACTCTGAACGCTAAAGTTG
P24	*lpp* D13P Q15P_R	CAACTTTAGCGTTCAGAGTCGGAACCGGAGAAGACAGCTGATCG
P25	*lolE* R239A_F	CAACGCCAATAAGCTGGTAGCCGATGCGGGTG
P26	*lolE* R239A_R	CACCCGCATCGGCTACCAGCTTATTGGCGTTG
P27	*lolE* Y248GY250G_F	GCGGGTGAAGTGACCAACAGCGGTGTTGGTATTAAAAGCTGGAT TGGTACTTAC
P28	*lolE* Y248GY250G_R	GTAAGTACCAATCCAGCTTTTAATACCAACACCGCTGTTGGTCAC TTCACCCGC
P29	*lolE* PP_F	GCGGGTGAAGTGACCAACAGCTATCCTTATCCTAAAAGCTGGAT TGGTACTTACGG
P30	*lolE* PP_R	CCGTAAGTACCAATCCAGCTTTTAGGATAAGGATAGCTGTTGGT CACTTCACCCGC
P31	*lolE* GPGP_F	GCGGGTGAAGTGACCAACAGCGGTCCTGGTCCTAAAAGCTGGAT TGGTAC
P32	*lolE* GPGP_R	GTACCAATCCAGCTTTTAGGACCAGGACCGCTGTTGGTCACTTCA CCCGC
P33	*lolE* R239C_F	CAACGCCAATAAGCTGGTATGCGATGCGGG
P34	*lolE* R239C_R	CCCGCATCGCATACCAGCTTATTGGCGTTG
P35	*lolE* Y250C_F	GAAGTGACCAACAGCTATGTTTGTATTAAAAGCTGGATTGGTAC

P36	*lolE* Y250C_R	GTACCAATCCAGCTTTTAATACAAACATAGCTGTTGGTCACTTC
P37	*lolE* I251C_F	GGTGAAGTGACCAACAGCTATGTTTATTGTAAAAGCTGGATTGG TAC
P38	*lolE* I251C_R	GTACCAATCCAGCTTTTACAATAAACATAGCTGTTGGTCACTTCA CC
P39	*lolE* Δcleft_F	CGGATGTTTTCAACGCCGGTAGCAGCTGGATTGGTACTTAC
P40	*lolE* Δcleft_R	GTAAGTACCAATCCAGCTGCTACCGGCGTTGAAAACATCCG
P41	*lolB* Δ6-15_F	CTGTTCCGTTACCACGCCACAATGGCGTCAGC
P42	*lolB* Δ6-15_R	GCTGACGCCATTGTGGCGTGGTAACGGAACAG
P43	*lolB* Δ9-15_F	GTTACCACGCCCAAAGGTCCACAATGGCGTCAG
P44	*lolB* Δ9-15_R	CTGACGCCATTGTGGACCTTTGGGCGTGGTAAC
P45	*lolB* Δ13-15_F	CCTGGCAAAAGCCCGCAATGGCGTCAGCATC
P46	*lolB* Δ13-15_R	GATGCTGACGCCATTGCGGGCTTTTGCCAGG

Table S4. List of plasmids.

Name	Description	Reference
pLDR8	*int* gene expression vector, helper plasmid	(8)
pLDR9	Cloning vector for integration into attB, kan resistant	(8)
pKD13	Kan cassette template for lambda red recombination	(9)
pSIM5	Expression of lambda red recombination genes	(10)
pET Duet	Expression vector	Novagen
pCDF Duet	Expression vector	Novagen
pCDF Lpp $_{\Delta 78\ Strep}$	Lpp with a ΔK58 mutation and a C-terminal strep tag	This study
pCDF Lpp $_{L10P\ \Delta 78\ Strep}$	Lpp with L10P ΔK58 mutation and a C-terminal strep tag	This study
pCDF Lpp$_{FL}$	Full length Lpp (untagged)	This study
pCDF Lpp$_{FL\ D13PQ15P}$	Full length Lpp with D13P+Q15P mutation (untagged)	This study
pCDF Pal$_{strep}$	Full length with C-terminal twin-strep tag	This study
pCDF LolB$_{strep}$	Full length LolB with C-terminal twin-strep tag	This study
pCDF LolB$_{Alfa}$	Full length LolB with C-terminal Alfa-tag	This study
pCDF LolB$_{\Delta X\text{-}Y\ Alfa}$	C-terminally Alfa-tagged LolB with residues X-Y inclusive deleted	This study
pET Duet LolCDE	LolCDE with C-terminal His-tag on LolD	(11)
pET Duet LolCDE XnY	pLolCDE with residue X mutated to residue Y at position n.	This study
pET Duet LolCDE$_{\Delta cleft}$	pLolCDE with LolE residues 235-252 replaced by a GS linker.	This study

Supplemental Methods

Strain and plasmid construction

Details of plasmids and the primers used to construct them are detailed in Tables S3 and S4. Strain HD200313 is a conditional knockout of MG1655 with an arabinose promoter replacing the native *lolCDE* promoter and is described in Tang *et al* 2021 (12). The chromosomal copy of *lpp* was knocked out from strain HD200313 using the λ Red recombinase system as described (9) except that following amplification of pKD13 with primers P1 and P2, pSIM5 (10) was used for recombinase expression. Deletions were confirmed by PCR of the gene locus with primers P3 and P4.

To construct an arabinose-inducible *lolB* conditional knockout strain (BW65), the *lolB* locus including the ribosome binding site was amplified using primers P5 and P6 and cloned into the NheI-HindIII sites of pBAD18 (13). The region encompassing the *araC* gene, pBAD promoter, *lolB* and the downstream terminator was amplified using primers P7 and P8, digested ClaI-SacI and cloned into the integration vector pLDR9 digested with the same enzymes. The construct was integrated into the lambda *attB* site of *E. coli* BW25113 according to a previously described protocol (8). The native copy of *lolB* was replaced with a kanamycin resistance cassette by amplifying pKD13 with primers P9 and P10 using the λ Red recombinase system as described (9) except that pSIM5 (10) was used for recombinase expression. Deletions were confirmed by PCR of the gene locus with primers P11 and P12.

Construction of pET Duet-LolCDE used for expression of His-tagged *E. coli* LolCDE has been described previously (11). To express Strep tagged Pal and LolB, a synthesised gene fragment (IDT DNA) containing the entire coding sequence with a C-terminal Strep-tag was amplified with primer pairs (P14/P15) and P14/P16) respectively and cloned into pCDF Duet digested with Nco/KpnI enzymes using Gibson assembly (14). For C-terminally strep-tagged Lpp, the coding sequence lacking the terminal lysine residue was amplified with primers P13 and P14 and cloned into pCDF Duet as described for the Pal and LolB constructs. To express wild-type Lpp a gene fragment was synthesised (IDT DNA) encoding full length wild-type untagged Lpp in which the native promoter was replaced with a constitutive promoter (TTGATGGCTAGCTCAGTCCTAGGTACAGTGCTAGC). This fragment was amplified with primer pair P17/P18 and introduced into EcoNI-KpnI digested pCDF Duet by Gibson assembly (14) resulting in pCDF Lpp_{FL}.

pCDF $LolB_{Alfa}$ was created by synthesizing a fragment encoding the full length *lolB* sequence with a C-terminal Alfa-tag (SRLEEELRRRLTE, (15) (IDT DNA). Following amplification with primers P19 and P20, the fragment was introduced into pCDF Duet digested Nco-KpnI using Gibson assembly (14). Subsequently residues 9-15 of the mature protein removed by Quikchange mutagenesis resulting in pCDF $LolB_{\Delta 9\text{-}15\ Alfa}$. Where required, point mutations in LolCDE or substrates were introduced using Quikchange mutagenesis (Agilent). All constructs were verified with DNA sequencing (Source Bioscience) prior to use.

Protein expression and purification

For all LolCDE and lipoprotein co-expressed constructs, chemically competent *E. coli* C43 cells (16) were transformed with pET Duet LolCDE (11) and the appropriate lipoprotein expression plasmid, and a seed culture grown in 2YT medium overnight at 37 °C. The next day, the seed culture was used to inoculate cultures in a LEX bioreactor (Epiphyte). Cells were grown to an OD_{600} ~3.6 at 37 °C before induction at 30 °C with 1 mM IPTG. Cells were harvested by centrifugation and stored frozen at -80 °C. Membranes were prepared by cell resuspension in Buffer A (25 mM HEPES pH 8.0, 150 mM NaCl, 5 mM $MgSO_4$, lysozyme, DNase, and 30% glycerol) before being passed twice through a cell disruptor (Constant Systems), at 32 000 psi. Cellular debris was removed by centrifugation at 10 000 RCF for 10 minutes before membranes were isolated by ultracentrifugation for 2 hours at 170 000 RCF and resuspended in Buffer A using a glass homogenizer to a final concentration of 0.67g/ml before cold storage at -80 °C.

Protein purification followed as 1% solubilization of membranes with Lauryl Maltose Neopentyl Glycol (LMNG, Anatrace) for 1 hour with rotation at 4 °C. Solubilized material was isolated by ultracentrifugation at 170 000 RCF for 1 hour at 4 °C, before being batch-bound on 1ml column volume of Promega IMAC resin (Biorad) with agitation for 1 hour at 4 °C. Beads were loaded onto a gravity column, washed with 30 column volumes of Buffer B (25mM HEPES pH 8.0, 150 mM NaCl, 30 mM Imidazole, 5 mM $MgSO_4$, 1 mM TCEP, 5% glycerol), 10 column volumes of Buffer C (25 mM HEPES pH 8.0, 1 M NaCl, 5 mM $MgSO_4$, 1 mM TCEP, 5% glycerol), 10 column volumes again of Buffer B, and finally eluted in 8 column volumes of Buffer D (25 mM HEPES 8.0, 150 mM NaCl, 300 mM Imidazole, 1 mM TCEP, 5 mM $MgSO_4$, 5% glycerol). Samples were than desalted by FPLC over a 26/10 column in Buffer E (25 mM HEPES pH 8.0, 150 mM NaCl, 1 mM TCEP, 5 mM $MgSO_4$). For

LolCDE+Pal_{strep}, protein was further purified by passage of desalted material 3 times over 1ml column volume of Strep-Tactin4Flow (IBA) at 4 °C, washed with 30 column volumes of Buffer F (25 mM HEPES pH 8.0, 150 mM NaCl, 5 mM $MgSO_4$), and eluted in 8 ml of 2x BXT elution buffer (200 mM TrisHCl pH 8.0, 300 mM NaCl, 2 mM EDTA, 100 mM Biotin) at 4 °C. For LolCDE+$LolB_{\Delta 9\text{-}15\ Alfa}$, desalted protein was passed 3 times over 1 ml of ALFA Selector CE resin (NanoTag) at 4 °C, washed with 30 column volumes in Buffer F, and eluted in 8 ml of Buffer F plus 200 µM ALFA elution peptide (NanoTag). Following affinity purification, all protein samples were concentrated to ~500 µl in a 100 kDa cutoff Amicon-Ultra 15 spin filter (Millipore) and injected onto a Superdex 200 10/300GL gel filtration column at 4 °C. 200µl fractions were collected and the mid-peak fraction was taken for subsequent cryo-EM analysis. ATPγS (Jena Biosciences) was added at a concentration of 5 mM to generate the closed state of the protein when required. To confirm the presence of substrate, purified complexes were resolved on 4-12% gradient SDS PAGE gels and transferred to PVDF membrane. Strep-tagged substrates were detected by immunoblotting with StrepMAB-Classic antibody (IBA) while anti-His (Qiagen) revealed His-tagged LolD. Following application of goat anti-mouse 800nm IR dye conjugated secondary antibody (LI-COR Biosciences), immunoblots were revealed using an Odyssey fluorescence imager (LI-COR Biosciences).

Cryo electron microscopy sample preparation, data collection and image processing

Quantifoil Cu 300 1.2/1.3 grids were treated by glow-discharge using a PELCO easiGLOW device with settings of 60 s, 25 mA, 0.39 mBar. For vitrification, 3 µL of sample was applied to the grids and the grids blotted with a blot force of -5 for 3 s using a Vitrobot Mark IV (ThermoFisher) at 4 °C and 95% humidity immediately before plunge-freezing in liquid ethane. Grids were subsequently stored in liquid nitrogen until screening and collection. Workflow for data acquisition is shown in Fig S5. Datasets were collected on a 300 kV Titan Krios equipped with a Gatan K3 or Falcon 4i detector. Data collection parameters are summarized in Table S1. Particles were picked and pre-processed (motion correction, CTF estimation and particle extraction) with WARP1.0.9. After migration of particle stacks to cryoSPARC (V4.0(17)) 2D classification, ab initio reconstruction, and multiple iterative rounds of heterogenous refinement were performed before 3D non-uniform refinement yielded the final reconstructions. In some cases, local refinements with soft masks around the area of interest were performed resulting in moderate improvements in visual map quality in these regions. All maps were anisotropically sharpened as implemented within PHENIX (4). The

locally refined and sharpened subassembly maps were combined to generate composite maps which were deposited to the EMDB.

Model building and refinement

Model building in COOT was aided by sharpened maps and composite maps of the highest available resolution (18, 19). The initial model for the LolCDE complex with lipoproteins was constructed using a combination of elements from previously published models. LolD was based on chain D from 7ARJ (12) with the dimer constructed by NCS fitting. Building of the first β-hairpin turn (residues 12-26) was guided by other ABC transporter structures identified by structural homology searches of the PDB using the SSM server: PDB efold (20).

Initial modelling of the LolC and LolE chains used PDB 7ARH and PDB 7ARI (12) broken into subdomains and rigid body real-space fitted in COOT (19). The register of the sequence was checked, and the fit was improved by rebuilding - particularly in the N-terminal section of LolE and in the periplasmic domain of LolE around Ser247 adjacent to the lipoprotein linker. The building of the LolC shoulder loop was partly guided by PDB 7MDX (21). In addition, the structurally homologous *Streptococcus pneumoniae* transporter, PDB 5XU1, (22) aided the rebuilding of the LolC loop 206-218. Models from a loop database search (in Swiss PDB Viewer (23)) were also used to guide the fitting of shoulder loops and LolC residues 15-22. Poor density attributed to the LolD C-terminal His-tag was observed in multiple maps but did not permit modelling of the tag.

The hetero atom groups for the lipoprotein acyl chain and linkage to Cys1 of the linker peptide were based on those in published PDBs including the LolCDE-complexed lipoprotein in PDB 7ARH (12) and *Pseudomonas* lipoprotein OprN PDB 5IUY (24). The acyl chains and associated moieties were initially fitted to the density using COOT (19) before real-space refinement in PHENIX with restraints generated in eLBOW (25, 26). Initial modelling of the extended lipoprotein linkers were also guided by known higher resolution extended loops (23). In general, all residues of linker sections were modelled in order to demonstrate the density, although weak, is consistent with the lengths of extended polypeptide given reasonable Ramachandran values. Real-space refinement and restrained ADP refinement used the standard protocol for EM data in PHENIX (25). Owing to the moderate resolution of the data, Ramachandran and secondary structure restraints were applied in addition to the standard geometric restraints. Rotameric restraints were also used to minimize outliers. The highest

resolution models for the LolC, LolD, and LolE chains (taken from their refined structures in complex with the $Lpp_{\Delta K58,L10P}$ linker) provided reference model dihedral restraints for the other complexes. In the final refinement cycles, non-crystallographic symmetry (NCS) restraints were not applied to the LolD chains. The secondary structure in all the final refined models was as defined by PHENIX - which makes allowance for refinement of moderate-resolution EM structures (25).

The dictionaries for the acyl groups were prepared in PHENIX using the eLBOW facility (26). Lipid linkage angles and distances for the bonds to the N and SG atoms of Cys1 were restrained based on similar small molecule examples. In addition, a planarity restraint was applied to the N-terminal linking N-C=O unit reflecting the partial double bond character of the amide bond. The folded domain of LolB was initially built using PDB 1IWM (27) but with rigid body rotation at its N-terminus to locate it in density and extend it through the linker density. The Pal domain was based on PDB 2W8B (28) also extended to fit the linker density - partly guided by the higher resolution structure PDB 4PWT (*Yersinia pestis* Pal) that has greater coverage of the amino-terminus of the domain. Statistics for structural alignments of LolCDE structures were generated using CCP4 Lsqkab (29) in the CCP4 suite (30).

In vivo growth assays of LolCDE variants

Strain HD200313 bearing empty vector, pET Duet LolCDE wild-type, or the indicated variant was cultured in LB medium supplemented with 100 µg/ml carbenicillin and 0.2% arabinose (to induce expression of the chromosomal wild-type copy of *lolCDE*). The next day, 1 mL of overnight culture was centrifuged at 4500 RCF for 2 mins, washed twice in LB and then serial tenfold dilutions performed in LB. Dilutions were plated out on LB agar with and without arabinose and grown overnight at 37 °C before imaging the next day. Where indicated 0.5 mM EDTA and 0.5% SDS were added to the medium to reveal a compromised cell envelope. For experiments involving *lpp* expression, strain HD200313 Δ*lpp* was cotransformed with pET Duet LolCDE wild-type or the indicated variant and pCDF Lpp_{FL} or D13P+Q15P variant and the assay performed as described above. Expression of Lpp was assessed from whole cell samples resolved on 4-12% gradient gels, transferred to PVDF membrane and revealed with anti-Lpp (Abbexa) and a 680 nm IR dye-conjugated anti-rabbit antibody (LI-COR Biosciences).

To verify expression of the $LolE_{\Delta cleft}$ variant, membranes were isolated from HD200313 cells bearing pET Duet LolCDE or the $LolCDE_{\Delta cleft}$ variant were grown in the presence of arabinose. LolCDE proteins were then purified as described above, were resolved on 12% SDS PAGE gels, transferred to PVDF and revealed with anti-LolE and a 680 nm IR dye-conjugated anti-rabbit antibody (LI-COR Biosciences). All immunoblots were imaged using an Odyssey fluorescence imager (LI-COR Biosciences).

To assess growth in liquid culture, strain HD200213 bearing empty vector, pET Duet LolCDE wild-type, or the indicated variant was grown overnight in LB supplemented with 100 µg/ml carbenicillin, 0.2% arabinose and then diluted 1/100 into the same medium lacking arabinose. Once the OD600 of these cultures had reached 0.4, cells were washed 3 times in LB and then 10 ul of culture added to 1ml of LB in a 24 well plate (Nunc). The plate was incubated at 37 °C and shaken at 200 rpm in a CLARIOstar Plus Plate reader (BMGlabtech) and the optical density at 600 nm recorded over 20 hours. The results presented are the average of at least 3 independent cultures.

In vivo **growth assays of LolB variants**

Strain BW65 bearing pCDF $LolB_{Alfa}$ or the indicated variant was cultured in LB medium supplemented with 0.2% arabinose and appropriate antibiotics. The next day, 1 mL of overnight culture was centrifuged at 4500 RCF for 2 mins, washed twice in LB supplemented with 0.1% D-fucose and then diluted 1/100 into fresh LB containing 0.1% fucose. Cells were then grown to an OD600 of 0.5, collected at 4500 *g*, washed twice in LB and then serial tenfold dilutions performed in LB medium. Dilutions were plated out on LB agar with and without arabinose and grown overnight at 37 °C before imaging the next day. Where indicated 0.5 mM EDTA and 0.5% SDS were added to the medium to reveal a compromised cell envelope. To assess expression level of the variant proteins, whole cell samples were resolved on 12% SDS-PAGE gels, transferred to PVDF membrane, and immunoblotted with a 680 nm IR dye conjugated anti-Alfa antibody (Nanotag). Immunoblots were revealed using an Odyssey fluorescence imager (LI-COR Biosciences).

E. coli **cell fractionation**

Strain BW65 bearing pCDF LolB or the indicated variant was cultured in 2YT medium at 37 °C until the OD_{600} reached 0.8. Cells were then harvested at 6000 RCF and resuspended in 50 mM Tris pH 8, 250 mM sucrose and lysozyme (0.15 mg/ml final concentration) added. An equal

volume of 1.5 mM EDTA pH 8 was then slowly added with mixing, and the cells incubated on ice for 10 minutes. Cells were then lysed in a cell disruptor (Constant Systems) at 20 000 p.s.i. Unbroken cells were removed by centrifugation for 10 minutes at 6000 RCF and the resultant supernatant centrifuged at 170 000 RCF for 2 hours. The resultant membrane pellet was resuspended in 25 mM HEPES pH7.5, 250 mM sucrose, 1 mM EDTA. To separate inner and outer membrane fractions, membranes were then loaded on a sucrose gradient consisting of 12 ml each of 0.77M, 1.44M, and 2.02 M sucrose in 25 mM HEPES pH7.5, 1 mM EDTA and centrifuged for 18 hours at 116 000 RCF. 1.5ml fractions were then collected and analysed on SDS PAGE, transferred to PVDF membrane and immunoblotted with antibodies against Lpp (Abbexa), SecG, or the Alfa-tag (Nanotag) antibodies. Immunoblots were revealed with dye conjugated anti-mouse secondary antibodies (LI-COR Biosciences) and revealed on an Odyssey scanner (LI-COR Biosciences). SecG and Lpp served as markers for the inner and outer membranes respectively. For wild-type LolB and variant proteins immunoblots from three independent fractionations were quantified using Image Studio software (LI-COR Biosciences) and the proportion of LolB protein in the outer membrane calculated.

Supplemental References

1. W. Qiao, *et al.*, Deciphering the molecular basis of lipoprotein recognition and transport by LolCDE. *Signal Transduct. Target. Ther.* **9**, 354 (2024).
2. A. Sueki, F. Stein, M. M. Savitski, J. Selkrig, A. Typas, Systematic Localization of *Escherichia coli* Membrane Proteins. *mSystems* **5** 00808-19 (2020).
3. G. E. Crooks, G. Hon, J. M. Chandonia, S. E. Brenner, WebLogo: A sequence logo generator. *Genome Res.* **14**, 1188–1190 (2004).
4. P. V. Afonine, *et al.*, Real-space refinement in PHENIX for cryo-EM and crystallography. *Acta Crystallogr. Sect. D Struct. Biol.* **74**, 531–544 (2018).
5. S. C. Lovell, *et al.*, Structure validation by Cα geometry: φ,ψ and Cβ deviation. *Proteins Struct. Funct. Genet.* **50**, 437–450 (2003).
6. I. W. Davis, *et al.*, MolProbity: All-atom contacts and structure validation for proteins and nucleic acids. *Nucleic Acids Res.* **35** W375–W383 (2007).
7. W. Bei, *et al.*, Cryo-EM structures of LolCDE reveal the molecular mechanism of bacterial lipoprotein sorting in *Escherichia coli*. *PLOS Biol.* **20**, e3001823 (2022).
8. L. Diederich, L. J. Rasmussen, W. Messer, New cloning vectors for integration into the λ attachment site attB of the *Escherichia coli* chromosome. *Plasmid* **28**, 14–24 (1992).
9. K. A. Datsenko, B. L. Wanner, One-step inactivation of chromosomal genes in *Escherichia coli* K-12 using PCR products. *Proc. Natl. Acad. Sci. U. S. A.* **97**, 6640–5 (2000).
10. S. Datta, N. Costantino, D. L. Court, A set of recombineering plasmids for gram-negative bacteria. *Gene* **379**, 109-115 (2006).
11. E. Kaplan, N. P. Greene, A. Crow, V. Koronakis, Insights into bacterial lipoprotein trafficking from a structure of LolA bound to the LolC periplasmic domain. *Proc. Natl. Acad. Sci. U. S. A.* **115**, E7389–E7397 (2018).

12. X. Tang, *et al.*, Structural basis for bacterial lipoprotein relocation by the transporter LolCDE. *Nat. Struct. Mol. Biol.* **28**, 347–355 (2021).
13. L. M. Guzman, D. Belin, M. J. Carson, J. Beckwith, Tight regulation, modulation, and high-level expression by vectors containing the arabinose P(BAD) promoter. *J. Bacteriol.* **177**, 4121–4130 (1995).
14. D. G. Gibson, *et al.*, Enzymatic assembly of DNA molecules up to several hundred kilobases. *Nat. Methods* **6**, 343–345 (2009).
15. H. Götzke, *et al.*, The ALFA-tag is a highly versatile tool for nanobody-based bioscience applications. *Nat. Commun.* **10** 4403 (2019).
16. B. Miroux, J. E. Walker, Over-production of Proteins in *Escherichia coli*: Mutant Hosts that Allow Synthesis of some Membrane Proteins and Globular Proteins at High Levels. *J. Mol. Biol.* **260**, 289–298 (1996).
17. A. Punjani, J. L. Rubinstein, D. J. Fleet, M. A. Brubaker, CryoSPARC: Algorithms for rapid unsupervised cryo-EM structure determination. *Nat. Methods* **14**, 290–296 (2017).
18. A. Casañal, B. Lohkamp, P. Emsley, Current developments in Coot for macromolecular model building of Electron Cryo-microscopy and Crystallographic Data. *Protein Sci.* **29**, 1069–1078 (2020).
19. P. Emsley, B. Lohkamp, W. G. Scott, K. Cowtan, Features and development of Coot. *Acta Crystallogr. Sect. D Biol. Crystallogr.* **66**, 486–501 (2010).
20. E. Krissinel, K. Henrick, Secondary-structure matching (SSM), a new tool for fast protein structure alignment in three dimensions. *Acta Crystallogr. D. Biol. Crystallogr.* **60**, 2256–68 (2004).
21. S. Sharma, *et al.*, Mechanism of LolCDE as a molecular extruder of bacterial triacylated lipoproteins. *Nat. Commun.* **12**, 4687 (2021).
22. H.-B. Yang, *et al.*, Structure of a MacAB-like efflux pump from *Streptococcus pneumoniae*. *Nat. Commun.* **9**, 196 (2018).
23. N. Guex, M. C. Peitsch, SWISS-MODEL and the Swiss-PdbViewer: An environment for comparative protein modeling. *Electrophoresis* **18**, 2714–2723 (1997).
24. Y. V. Ntsogo Enguéné, *et al.*, Xenon for tunnelling analysis of the efflux pump component OprN. *PLoS One* **12**, e0184045 (2017).
25. D. Liebschner, *et al.*, Macromolecular structure determination using X-rays, neutrons and electrons: Recent developments in PHENIX. *Acta Crystallogr. Sect. D Struct. Biol.* **75**, 861–877 (2019).
26. N. W. Moriarty, R. W. Grosse-Kunstleve, P. D. Adams, Electronic ligand builder and optimization workbench (eLBOW): A tool for ligand coordinate and restraint generation. *Acta Crystallogr. Sect. D Biol. Crystallogr.* **65**, 1074–1080 (2009).
27. K. Takeda, *et al.*, Crystal structures of bacterial lipoprotein localization factors, LolA and LolB. *EMBO J.* **22**, 3199–209 (2003).
28. D. A. Bonsor, *et al.*, Allosteric Β-propeller signalling in TolB and its manipulation by translocating colicins. *EMBO J.* **28**, 2846–2857 (2009).
29. W. Kabsch, A solution for the best rotation to relate two sets of vectors. *Acta Cryst.* **A32**, 922–923 (1976).
30. J. Agirre, *et al.*, The CCP4 suite: integrative software for macromolecular crystallography. *Acta Crystallogr D Struct Biol* **79**, 449–461 (2023).
